# Comparative efficacy of dietary interventions and enteral nutrition in adults with ulcerative colitis: a network meta-analysis

**DOI:** 10.3389/fnut.2026.1886706

**Published:** 2026-08-25

**Authors:** Tingting Wang, Ling Ji, Chunyan Sun, Hui Liu, Jian Li, Yanyan Hong

**Affiliations:** 1 Department of Proctology, Nanjing Hospital of Chinese Medicine Affiliated to Nanjing University of Chinese Medicine, Nanjing, China; 2 Guangdong Mental Health Center, Guangdong Provincial People’s Hospital, Guangzhou, China; 3 Department of Nursing, Nanjing Hospital of Chinese Medicine Affiliated to Nanjing University of Chinese Medicine, Nanjing, China

**Keywords:** dietary therapy, enteral nutrition, network meta-analysis, nutritional support, ulcerative colitis

## Abstract

**Objective:**

To compare the effects of dietary and enteral nutrition interventions on clinical effectiveness, albumin, C reactive protein, and quality of life in adults with ulcerative colitis.

**Methods:**

PubMed, Web of Science, Embase, CNKI, SinoMed, and Wanfang were searched from inception to January 2026. Randomized controlled trials evaluating dietary or enteral nutrition interventions in adults with ulcerative colitis were included. Bayesian network meta analysis was performed using odds ratios or mean differences with 95% credible intervals. Risk of bias and confidence in the evidence were assessed using RoB 2 and CINeMA.

**Results:**

Fourteen trials involving 1,039 participants and 10 interventions were included. Enteral nutrition was associated with greater clinical effectiveness than a low residue diet (OR 4.80, 95% CrI 1.21–23.52), and the finding remained consistent in sensitivity analyses. Compared with a low residue diet, enteral nutrition, an IgG guided exclusion diet, a low-FODMAP diet, and a regular diet were associated with higher albumin levels. No statistically significant differences were found for quality of life or C-reactive protein. Confidence in all network estimates was low or very low.

**Conclusion:**

Enteral nutrition may improve clinical effectiveness compared with a low residue diet, while several interventions may increase albumin levels. However, the limited evidence precludes firm conclusions. Further large and rigorously designed randomized controlled trials are required.

**Systematic review registration:**

https://www.crd.york.ac.uk/PROSPERO/view/CRD420251076943.

## 1 Introduction

Ulcerative colitis (UC) is recognized as a major subtype of inflammatory bowel disease (IBD) (1). The disease typically follows a chronic and relapsing course, with common manifestations including diarrhea, rectal bleeding, abdominal pain, and tenesmus. These symptoms may progressively impair patients’ nutritional status, health-related quality of life, and social functioning (2). Globally, the prevalence of UC is estimated to be approximately 120.4 cases per 100,000 individuals, and the condition contributes to more than half of the total disease burden attributable to IBD (3). Furthermore, evidence from global epidemiological research indicates that the incidence of UC has been increasing in newly industrialized regions since 2000. This trend suggests that IBD is no longer confined mainly to Western populations but has become an important chronic health challenge worldwide (4).

Current treatment of UC mainly includes 5-aminosalicylic acid preparations, corticosteroids, immunosuppressants, biologic agents, and targeted oral therapies. However, these treatments still have important limitations, as some patients respond inadequately or develop secondary loss of response (5). Long term drug therapy may also cause adverse effects. Prolonged corticosteroid use is associated with diabetes, hypertension, infection, osteoporosis, and mood disorders (6), whereas thiopurines such as azathioprine carry risks of bone marrow suppression, hepatotoxicity, and lymphoma, while JAK inhibitors may be linked to a higher likelihood of major adverse cardiovascular events and malignant tumors (7). Therefore, identifying safer adjunctive strategies that are suitable for long term use, have fewer adverse effects, and can also improve symptom control, inflammation, and nutritional status has become an important focus in UC research.

Dietary factors play an important role in the pathogenesis and progression of UC and represent one of the most modifiable aspects of disease management. Previous studies suggest that dietary and nutritional interventions may influence intestinal inflammation through effects on gut microbiota, intestinal barrier function, metabolite production, and immune inflammatory responses. Accordingly, dietary therapy in UC is relevant not only to symptom control but also to inflammation remission and nutritional support (5). Current guidelines and expert consensus, including those from the American Gastroenterological Association (AGA) (8), emphasize the importance of nutritional management in comprehensive IBD care. However, no single dietary pattern has been consistently shown to universally reduce disease activity or relapse risk in adults with IBD (8). As evidence has accumulated, enteral nutrition (EN) (9), the low-FODMAP diet (LFD) (10), the Mediterranean diet (MD) (11), the gluten free diet (GFD) (12), and multimodal combined dietary interventions (13, 14) have all been investigated in UC, with potential benefits reported for clinical remission, inflammatory markers, nutritional status, and quality of life.

Nevertheless, several key gaps remain in the existing evidence. Previous studies (15) have performed network meta analyses of dietary interventions in broader IBD populations, and others (16) have focused on children with active Crohn’s disease, but these findings cannot be directly extrapolated to adults with UC. Moreover, existing reviews of UC (9, 14) have mainly used traditional systematic review or meta-analysis methods and have focused on a single dietary pattern, limiting systematic comparison and relative ranking of multiple dietary interventions within a unified framework. Thus, we conducted a network meta-analysis to systematically compare different dietary and nutritional interventions for adults with UC, aiming to generate more targeted evidence for clinical dietary therapy and nutritional management.

## 2 Methods

### 2.1 Study design and protocol registration

This study was designed as a network meta-analysis and was prospectively registered in PROSPERO under the registration number CRD420251076943. The reporting process followed the PRISMA extension statement for systematic reviews incorporating network meta-analysis (17).

### 2.2 Inclusion and exclusion criteria

(1) Participants were eligible if they were adults aged ≥18 years and had a confirmed diagnosis of UC in accordance with the British Society of Gastroenterology (BSG) guidelines (18), supported by clinical presentation, inflammatory biomarkers, endoscopic assessment, and histopathological confirmation (19). (2) The intervention group received a structured dietary intervention or enteral nutritional therapy, including dietary pattern modification, exclusion diets, enteral nutrition, partial enteral nutrition, and other related dietary strategies. (3) The control group was treated with an alternative dietary strategy or a standard diet. (4) Studies were required to report at least one of the following primary outcomes: ①. Clinical effectiveness; ②. Inflammatory Bowel Disease Questionnaire (IBDQ); ③. C-reactive protein (CRP); ④. Albumin (ALB); ⑤. Fecal calprotectin (FCP).

Studies were excluded if they met any of the following criteria: (1) publication in a language other than Chinese or English; (2) duplicate publication; (3) non-randomized controlled trial design; (4) animal studies; or (5) conference abstracts with insufficient methodological or outcome data were excluded.

### 2.3 Search strategy

We systematically searched PubMed, Web of Science, Embase, CNKI, SinoMed, and Wanfang from their inception through January 2026, without applying limits on publication year. To ensure completeness, we also examined the reference lists of the included studies and related reviews for additional records. The full search strategy is presented in Supplementary material.

### 2.4 Data extraction and synthesis

All identified records were first imported into EndNote 21 for duplicate removal. Subsequently, two reviewers independently screened the studies and extracted data using a standardized Excel spreadsheet developed by the research team. Extracted information included the first author, publication year, sample size, participant age range, intervention duration, intervention characteristics, and reported outcomes. Any disagreements were resolved by discussion or, when consensus could not be reached, by involving a third reviewer.

### 2.5 Quality assessment

Risk of bias across the included studies was evaluated with the Cochrane RoB 2 tool (20). The assessment focused on five methodological domains, namely randomization and allocation concealment, blinding of participants and investigators, incomplete outcome data, outcome measurement and reporting, and selective reporting of results. For each domain, studies were judged as “low risk,” “high risk,” or having “some concerns.” The assessments were conducted independently by two reviewers, with disagreements settled by discussion with a third reviewer.

### 2.6 Assessment of transitivity

The transitivity assumption was assessed qualitatively within each evidence network. Eligible interventions were limited to dietary and enteral nutrition strategies delivered through the gastrointestinal tract as adjunctive treatments for adults with UC. Despite differences in composition and implementation, they were considered comparable within a broad nutritional framework, without assuming biological equivalence or clinical interchangeability. Potential effect modifiers, including disease severity, treatment phase, baseline nutritional status, concomitant medications, and intervention duration, were examined alongside differences in disease activity measures and definitions of clinical remission when interpreting indirect comparisons and treatment rankings.

### 2.7 Statistical analysis

Continuous outcomes were summarized as mean differences (MDs), while dichotomous outcomes were expressed as odds ratios (ORs), each with 95% credible intervals (CrIs). Evidence networks were visualized using Stata 18.0, and Bayesian network meta analyses were performed in ADDIS using random effects consistency models. A binomial likelihood with a logit link was applied to clinical effectiveness, whereas normal likelihoods with identity links were used for ALB, IBDQ, and CRP. Four Markov chain Monte Carlo chains were run with 20,000 tuning iterations and 100,000 simulation iterations. A thinning interval of 10 and a variance scaling factor of 2.5 were applied, yielding 20,000 inference samples. Convergence was evaluated using the Brooks Gelman Rubin method, and potential scale reduction factor values close to 1 indicated satisfactory convergence. Because no closed loops were identified, inconsistency could not be formally assessed. Treatment rankings were summarized using SUCRA values, with higher values indicating a greater probability of a favorable ranking. Sensitivity analyses were performed by excluding studies at high risk of bias or with very small sample sizes when the evidence network remained connected. Very small studies were defined as those with fewer than 30 participants (21). Potential small study effects were assessed using comparison adjusted funnel plots when feasible.

### 2.8 Certainty of evidence assessment

The certainty of evidence for each network meta-analysis estimate was evaluated using the Confidence in Network Meta-Analysis (CINeMA) framework. The assessment covered six domains: within-study bias, reporting bias, indirectness, imprecision, heterogeneity, and incoherence. Judgements across these domains were integrated to assign an overall confidence rating of high, moderate, low, or very low to each network estimate (22, 23).

## 3 Results

### 3.1 Search results

**Figure 1:**
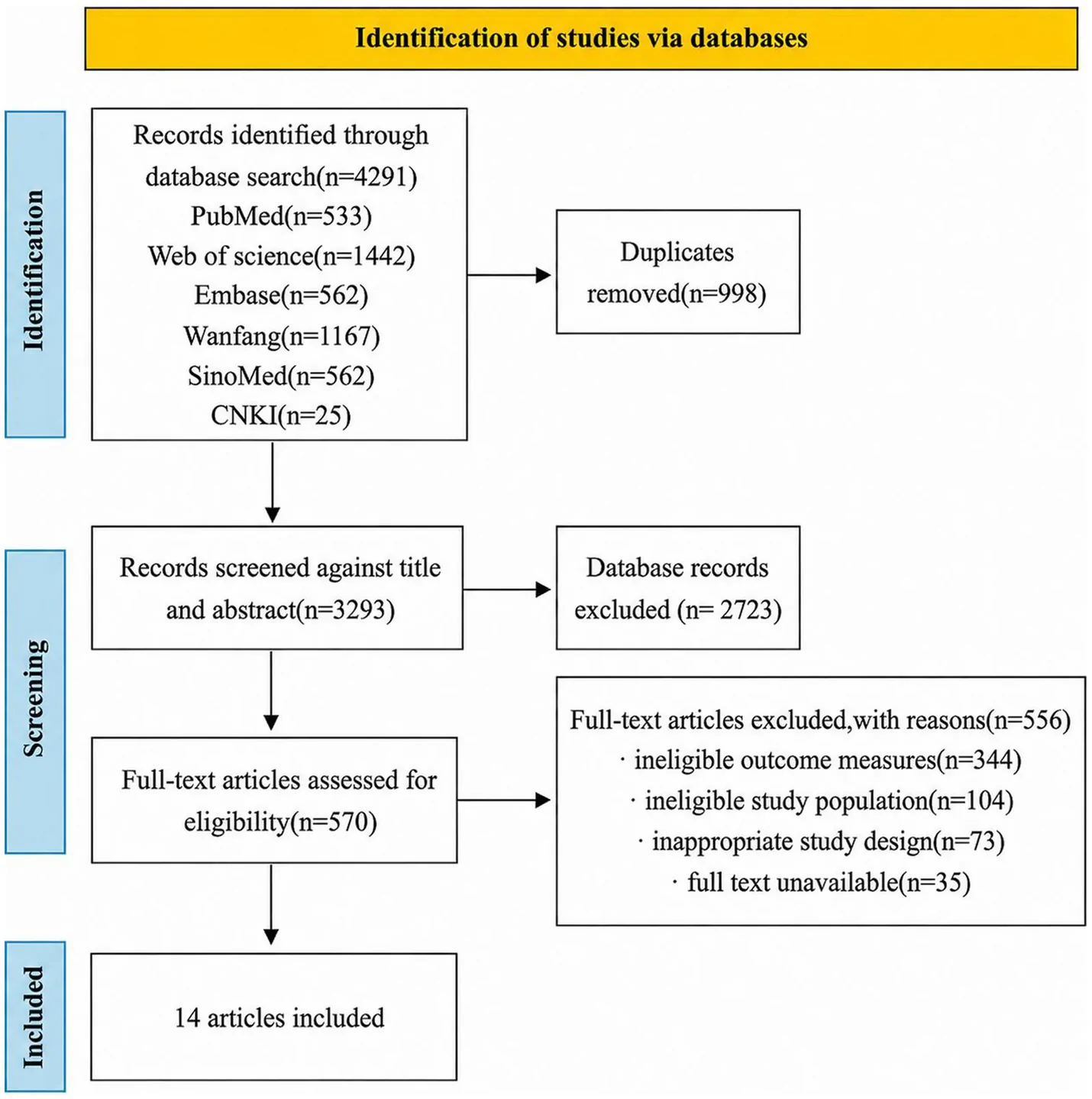
PRISMA flow diagram.

A total of 4,291 records were identified through the database search. Following the removal of 998 duplicates, 3,293 records were screened based on titles and abstracts. Following the screening of titles and abstracts, 2,723 records were removed, leaving 570 full-text articles for eligibility assessment. Of these, 14 studies (12, 13, 24–35) satisfied the predefined inclusion criteria and were finally incorporated into the meta-analysis. The process of study identification and selection is illustrated in the PRISMA flow diagram (Figure. 1).

### 3.2 Summary of included study characteristics

The analysis included 14 studies (12, 13, 24–35) with 1,039 participants, of whom 523 received the intervention and 516 were included in the control group. A total of 10 dietary interventions were assessed: regular diet (RD), Mediterranean diet (MD), low-residue diet (LRD), enteral nutrition (EN), partial enteral nutrition (PEN), specific carbohydrate diet (SCD), low-FODMAP diet (LFD), IgG-guided exclusion diet (IgG-ED), gluten-free diet (GFD), and traditional Chinese medicine diet (TCMD). Detailed baseline and methodological characteristics of the included studies are presented in Table 1. Intervention duration ranged from 21 days to 6 months across induction, maintenance, and mixed treatment settings. Baseline nutritional status was often incompletely reported, while concomitant therapy varied across trials but was generally balanced between groups within individual studies. Mesalazine was most commonly used, although corticosteroids, enemas, probiotics, traditional treatments, and other supportive therapies were permitted in some trials. The distribution of these potential effect modifiers across studies and direct treatment comparisons is summarized in Supplementary Table S1.

**Table 1:** Characteristics of included studies.

Author	Year	Disease Stage	Intervention characteristics		Sample size (n)		Sex (male)		Age (years)		Intervention time		Outcomes
			T	C	T	C	T	C	T	C	T	C	
Behnaz Narimani	2024	Moderate	MD + LFD + PEN	RD	25	25	10	13	34.88 ± 9.53	39.76 ± 12.46	6 weeks	6 weeks	②③
Long Xue	2024	Light, Moderate	EN	LRD	42	42	22	23	42.13 ± 2.37	41.31 ± 2.32	21 days	21 days	①
Foroogh Alborzi Avanaki	2024	Light, Moderate	GFD	RD	13	13	5	2	38.92 ± 9.94	39.69 ± 9.09	6 weeks	6 weeks	①③
ShengYin Chen	2024	Light, Moderate	SCD	MD	8	9	3	5	38.38 ± 15.77	44.22 ± 18.31	6 weeks	6 weeks	⑤
Natasha Haskey	2022	Light, Moderate	MD	RD	15	13	6	4	18–65	25–63	12 weeks	12 weeks	⑥
Jian Liu	2018	Light, Moderate	IgG-ED	RD	49	48	26	24	38(21–65)	39(20–68)	6 months	6 months	④
Limin Xin	2014	Light, Moderate, Severe	EN	RD	23	22	12	11	44 ± 16	46 ± 14	4 weeks	4 weeks	①
Zhe Chen	2024	Moderate, Severe	EN	LRD	58	58	28	36	45.85 ± 7.32	43.31 ± 10.04	6 weeks	6 weeks	①④
Weiguang Wang	2021	Light, Moderate	LFD	RD	63	66	27	31	44.52 ± 11.53	45.32 ± 11.73	6 weeks	6 weeks	②④
Xiaoyan Zhang	2020	Light, Moderate	LFD	RD	35	32	16	18	51.44 ± 9.91	52.19 ± 10.05	6 weeks	6 weeks	①③
Mei Yang	2024	Light, Moderate	LFD	RD	68	66	38	31	40.18 ± 5.12	39.77 ± 6.22	12 weeks	12 weeks	④
Yan Chen	2021	Light, Moderate	LFD	RD	35	32	16	18	48.60 ± 10.51	45.28 ± 10.16	6 weeks	6 weeks	①③④
Xianhong Han	2015	Light, Moderate	TCMD	RD	30	32	13	17	40.23 ± 9.84	37.53 ± 10.24	6 months	6 months	①②
Junwei Chen	2025	Light, Moderate, Severe	LFD	EN	59	58	28	25	45.31 ± 11.74	44.51 ± 11.52	6 weeks	6 weeks	④

T, treatment group; C, control group; −, not mentioned. RD, regular diet; MD, Mediterranean diet; LRD, low-residue diet; EN, enteral nutrition; PEN, Partial enteral nutrition; SCD, specific carbohydrate diet; LFD, low-FODMAP diet; IgG-ED, IgG-guided exclusion diet; GFD, Gluten-free diet; TCMD, traditional Chinese medicine diet. ①. clinical effectiveness; ②. IBDQ; ③. CRP; ④. ALB; ⑤. FCP, Fecal calprotectin; ⑥. SIBDQ (bowel domain).

### 3.3 Quality assessment

**Figure 2:**
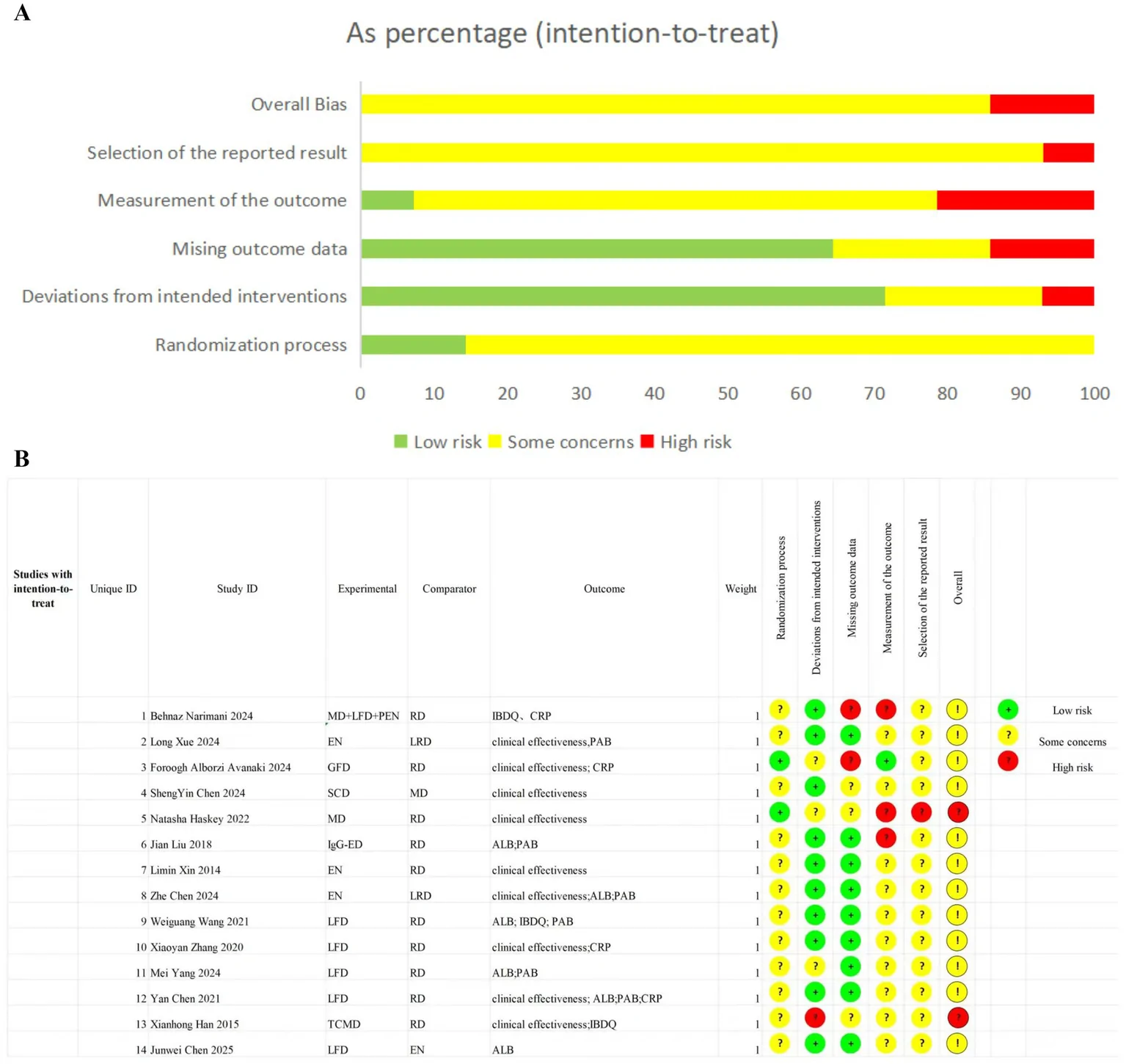
Bias risk assessment of the included randomized controlled trials. **(A)** Overall distribution of studies across different bias risk categories. **(B)** Domain level bias risk judgments for each individual study.

All 14 included studies (12, 13, 24–35) reported comparable baseline characteristics between groups. Two studies (12, 26) provided relatively detailed descriptions of the randomization methods, whereas the remaining 12 mentioned randomization but did not report key details such as allocation concealment. For bias arising from deviations from intended interventions, 10 studies were rated as low risk (13, 24, 25, 27–31, 33, 35). For missing outcome data, 9 studies (24, 27–33, 35) had complete outcome data, while 2 were rated as high risk because of substantial missingness (12, 13). Only 1 study (12) was judged as low risk for outcome measurement, and 10 studies (24, 25, 28–35) were rated as having some concerns, largely because blinding was difficult to implement in dietary interventions and several primary outcomes were relatively subjective. In addition, 13 studies (12, 13, 24, 25, 27–35) were judged as having some concerns for selective reporting because trial registration or a prespecified statistical analysis plan was unavailable. Overall, 12 studies were judged as having some concerns and two as being at high risk of bias (Figure 2).

### 3.4 Network meta-analysis results

#### 3.4.1 Evidence network plot

**Figure 3:**
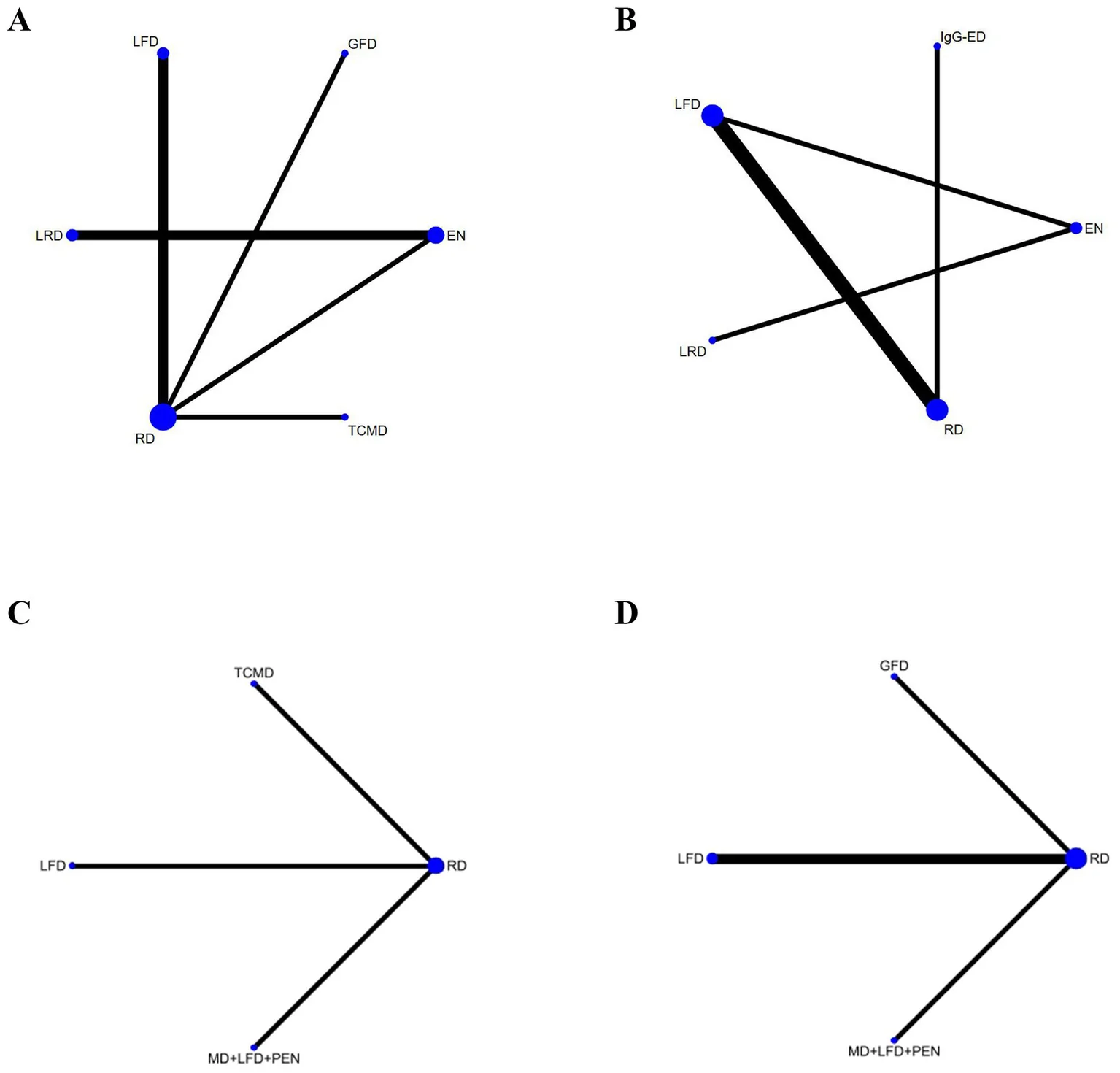
Network map. **(A)** Clinical effectiveness. **(B)** ALB. **(C)** IBDQ. **(D)** CRP. RD, regular diet; MD, Mediterranean diet; LRD, low-residue diet; EN, enteral nutrition; PEN, partial enteral nutrition; SCD, specific carbohydrate diet; LFD, low-FODMAP diet; IgG-ED, IgG-guided exclusion diet; GFD, gluten-free diet; TCMD, traditional Chinese medicine diet.

Among the 14 included studies, 10 dietary and nutritional interventions and 6 outcome indicators were identified. Two outcomes, FCP (25) and SIBDQ (26) were each reported in only one study and therefore could not be included in the network meta-analysis. Among the included studies, 7 studies (12, 24, 28, 29, 31, 33, 34) reported clinical effectiveness, 3 studies (13, 30, 34) reported IBDQ, 4 studies (12, 13, 31, 33) reported CRP, and 6 studies (27, 29, 30, 32, 33, 35) reported ALB (Figure 3).

#### 3.4.2 Clinical effectiveness

Clinical effectiveness was analyzed as a composite dichotomous outcome comprising complete or partial remission, clinical response, and overall effectiveness as defined in the original studies. Where specified, clinical response required a decrease in the modified Mayo score of at least 3 points and 30% from baseline, together with a decrease of at least 1 point in the rectal bleeding subscore (29). Given the variability in outcome definitions, the pooled findings were interpreted as exploratory. Seven studies (12, 24, 28, 29, 31, 33, 34) assessed clinical effectiveness across six dietary and enteral nutrition interventions. The network meta-analysis indicated that EN was associated with significantly higher odds of clinical effectiveness than LRD (OR = 4.80, 95% CrI: 1.21–23.52). No statistically significant differences were identified in the remaining pairwise comparisons (Table 2). The definitions of clinical effectiveness varied across studies and are summarized in Supplementary Table S2.

**Table 2:** Relative effects of different dietary patterns on clinical effectiveness.

EN					
7.53 (0.14, 496.30)	GFD				
3.67 (0.18, 168.68)	0.55 (0.03, 12.48)	LFD			
**4.80 (1.21, 23.52)**	0.65 (0.01, 47.34)	1.38 (0.02, 39.87)	LRD		
11.55 (0.87, 427.06)	1.71 (0.13, 29.69)	3.20 (0.69, 15.99)	2.39 (0.12, 109.51)	RD	
9.04 (0.35, 513.84)	1.27 (0.05, 45.64)	2.46 (0.20, 30.97)	1.94 (0.05, 135.00)	0.76 (0.10, 5.74)	TCMD

Each estimate represents the column-defined intervention versus the row-defined intervention. An OR greater than 1 favours the column defining intervention, whereas an OR less than 1 favours the row defining intervention.

Statistically significant estimates are highlighted in bold.

EN, enteral nutrition; GFD, gluten-free diet; LFD, low-FODMAP diet; LRD, low-residue diet; RD, regular diet; TCMD, traditional Chinese medicine diet.

#### 3.4.3 ALB

A total of six studies (27, 29, 30, 32, 33, 35) evaluated ALB levels across five dietary and enteral nutrition interventions. Compared with LRD, EN, IgG-ED, LFD, and RD were associated with significantly higher ALB levels, with MDs of 4.11 (95% CrI: 0.48–7.71), 8.84 (95% CrI: 2.07–15.32), 7.26 (95% CrI: 2.04–12.35), and 6.49 (95% CrI: 0.77–11.91), respectively. No statistically significant differences were observed in the remaining pairwise comparisons (Table 3).

**Table 3:** Relative effects of different dietary patterns on ALB.

EN				
−4.81 (−10.17, 0.96)	IgG-ED			
−3.16 (−6.80, 0.51)	1.64 (−2.75, 5.68)	LFD		
**4.11 (0.48, 7.71)**	**8.84 (2.07, 15.32)**	**7.26 (2.04, 12.35)**	LRD	
−2.44 (−6.42, 1.84)	2.38 (−1.42, 6.06)	0.73 (−1.13, 2.89)	***−6.49 (−11.91, −0.77)***	RD

Each estimate represents the column-defined intervention versus the row-defined intervention. An MD greater than 0 favours the row defining intervention, whereas an MD less than 0 favours the column defining intervention.

Statistically significant estimates are shown in bold.

EN, enteral nutrition; IgG-ED, IgG-guided exclusion diet; LFD, low-FODMAP diet; LRD, low-residue diet; RD, regular diet.

#### 3.4.4 IBDQ

Three studies (13, 30, 34) reported IBDQ scores across four dietary and enteral nutrition interventions: LFD, MD combined with LFD and PEN, RD, and TCMD. The network meta-analysis found no statistically significant differences among the interventions, as all 95% credible intervals included zero. Although the point estimates favored RD and TCMD over LFD and the combined MD, LFD, and PEN intervention, the uncertainty surrounding these estimates precluded firm conclusions (Table 4).

**Table 4:** Relative effects of different dietary and nutritional interventions on IBDQ.

LFD			
−3.40 (−25.84, 19.31)	MD + LFD + PEN		
9.23 (−6.63, 25.26)	12.66 (−3.76, 28.80)	RD	
8.00 (−14.57, 29.98)	11.23 (−11.46, 33.20)	−1.43 (−16.56, 14.48)	TCMD

Each estimate represents the column-defined intervention versus the row-defined intervention. MD values above 0 favour the row defining intervention, while MD values below 0 favour the column defining intervention.

LFD, low-FODMAP diet; MD+LFD+PEN, Mediterranean diet combined with low-FODMAP diet and partial enteral nutrition; RD, regular diet; TCMD, traditional Chinese medicine diet.

#### 3.4.5 CRP

A total of four studies (12, 13, 31, 33) reported CRP levels across four dietary: GFD, LFD, MD combined with LFD and PEN, and RD. The network meta-analysis found no statistically significant differences among the interventions, as all 95% credible intervals included zero. Although the point estimates numerically favored GFD over LFD, the combined MD, LFD, and PEN intervention, and RD for reducing CRP levels, the uncertainty surrounding these estimates precluded firm conclusions (Table 5).

**Table 5:** Relative effects of different dietary and nutritional interventions on CRP.

GFD			
3.41 (−1.39, 8.31)	LFD		
3.03 (−2.20, 8.38)	−0.37 (−4.16, 3.45)	MD + LFD + PEN	
2.09 (−2.27, 6.41)	−1.34 (−3.55, 0.92)	−0.96 (−4.05, 2.11)	RD

Each estimate represents the column-defined intervention versus the row-defined intervention. Because lower CRP levels indicate a more favorable outcome, MD values above 0 favor the column-defined intervention, whereas MD values below 0 favor the row-defined intervention.

GFD, gluten-free diet; LFD, low-FODMAP diet; MD+LFD+PEN, Mediterranean diet combined with low-FODMAP diet and partial enteral nutrition; RD, regular diet.

#### 3.4.6 Ranking of dietary interventions based on SUCRA

The SUCRA based rankings are presented in Table 6. For clinical effectiveness, EN had the highest SUCRA value (90.60%), followed by LFD (63.20%). For ALB levels, IgG-ED ranked highest (91.75%), followed by LFD (75.00%). For improvement in IBDQ scores, the combined MD, LFD, and PEN intervention had the highest SUCRA value (84.67%), followed by LFD (68.67%). For CRP reduction, GFD ranked highest (88.00%), followed by RD (61.67%), the combined MD, LFD, and PEN intervention (32.00%), and LFD (18.67%).

**Table 6:** Ranking of intervention effectiveness.

Intervention	Clinical effectiveness		ALB		IBDQ		CRP	
	SUCRA (%)	Rank	SUCRA (%)	Rank	SUCRA (%)	Rank	SUCRA (%)	Rank
RD	21.41	6	52.00	3	16.67	4	61.67	2
LRD	48.08	3	1.00	5	–	–	–	–
EN	90.60	1	28.50	4	–	–	–	–
LFD	63.20	2	75.00	2	68.67	2	18.67	4
IgG-ED	–	–	91.75	1	–	–	–	–
GFD	42.57	4	–	–	–	–	88.00	1
TCMD	34.14	5	–	–	30.00	3	–	–
MD + LFD + PEN	–	–	–	–	84.67	1	32.00	3

ALB, albumin; CRP, C-reactive protein; IBDQ, Inflammatory Bowel Disease Questionnaire; EN, enteral nutrition; GFD, gluten-free diet; IgG-ED, IgG-guided exclusion diet; LFD, low-FODMAP diet; LRD, low-residue diet; MD, Mediterranean diet; PEN, partial enteral nutrition; RD, regular diet; TCMD, traditional Chinese medicine diet.

These rankings indicate the relative likelihood of each intervention achieving a favorable position within the evidence network. However, they should be interpreted with caution, particularly for IBDQ and CRP, because no statistically significant differences were identified in the pairwise comparisons. Accordingly, the SUCRA results should be regarded as exploratory and hypothesis generating rather than as conclusive evidence of treatment superiority.

#### 3.4.7 Confidence in the network estimates

Confidence in the network estimates was evaluated using CINeMA. Of the 37 pairwise comparisons assessed across the four outcomes, 16 were rated as having low confidence and 21 as having very low confidence. Downgrading was primarily driven by concerns regarding within-study bias, reporting bias, and indirectness. Imprecision substantially affected several comparisons, particularly for clinical effectiveness, while heterogeneity contributed to downgrading in relatively few cases (Table 7).

**Table 7:** Confidence ratings of network meta-analysis estimates assessed using CINeMA.

Outcome	Comparison	Confidence rating
Clinical effectiveness	EN vs. LRD	Low
Clinical effectiveness	EN vs. RD	Very low
Clinical effectiveness	GFD vs. RD	Very low
Clinical effectiveness	LFD vs. RD	Very low
Clinical effectiveness	RD vs. TCMD	Very low
Clinical effectiveness	EN vs. GFD	Very low
Clinical effectiveness	EN vs. LFD	Very low
Clinical effectiveness	EN vs. TCMD	Very low
Clinical effectiveness	GFD vs. LFD	Very low
Clinical effectiveness	GFD vs. LRD	Very low
Clinical effectiveness	GFD vs. TCMD	Very low
Clinical effectiveness	LFD vs. LRD	Very low
Clinical effectiveness	LFD vs. TCMD	Very low
Clinical effectiveness	LRD vs. RD	Very low
Clinical effectiveness	LRD vs. TCMD	Very low
ALB	EN vs. LFD	Low
ALB	EN vs. LRD	Low
ALB	IgG-ED vs. RD	Low
ALB	LFD vs. RD	Low
ALB	EN vs. IgG-ED	Low
ALB	EN vs. RD	Low
ALB	IgG-ED vs. LFD	Very low
ALB	IgG-ED vs. LRD	Low
ALB	LFD vs. LRD	Low
ALB	LRD vs. RD	Low
IBDQ	LFD vs. RD	Low
IBDQ	MD + LFD + PEN vs. RD	Low
IBDQ	RD vs. TCMD	Low
IBDQ	LFD vs. MD + LFD + PEN	Low
IBDQ	LFD vs. TCMD	Low
IBDQ	MD + LFD + PEN vs. TCMD	Low
CRP	GFD vs. RD	Very low
CRP	LFD vs. RD	Very low
CRP	MD + LFD + PEN vs. RD	Very low
CRP	GFD vs. LFD	Very low
CRP	GFD vs. MD + LFD + PEN	Very low
CRP	LFD vs. MD + LFD + PEN	Very low

Confidence ratings were assessed using CINeMA across six domains: within-study bias, reporting bias, indirectness, imprecision, heterogeneity, and incoherence. The direction of the comparison does not affect the overall confidence rating.

ALB, albumin; CRP, C-reactive protein; IBDQ, Inflammatory Bowel Disease Questionnaire; EN, enteral nutrition; GFD, gluten-free diet; IgG-ED, IgG-guided exclusion diet; LFD, low-FODMAP diet; LRD, low-residue diet; MD, Mediterranean diet; PEN, partial enteral nutrition; RD, regular diet; TCMD, traditional Chinese medicine diet.

#### 3.4.8 Sensitivity analyses

Sensitivity analyses were conducted when sufficient studies were available and the corresponding evidence networks remained connected. For clinical effectiveness, the results remained consistent with the primary analysis after excluding studies with a high overall risk of bias or very small sample sizes. EN continued to show higher odds of clinical effectiveness than LRD after excluding studies with a high overall risk of bias (OR = 4.99, 95% CrI: 1.24–22.73) and after excluding very small studies (OR = 4.81, 95% CrI: 1.29–21.61). No additional statistically significant differences were identified.

For CRP, excluding very small studies did not materially alter the findings. Sensitivity analyses could not be performed for ALB or IBDQ because study exclusion would have disconnected the evidence networks. Analyses stratified by disease severity were not feasible because subgroup specific data could not be extracted from the original studies.

### 3.5 Publication bias

**Figure 4:**
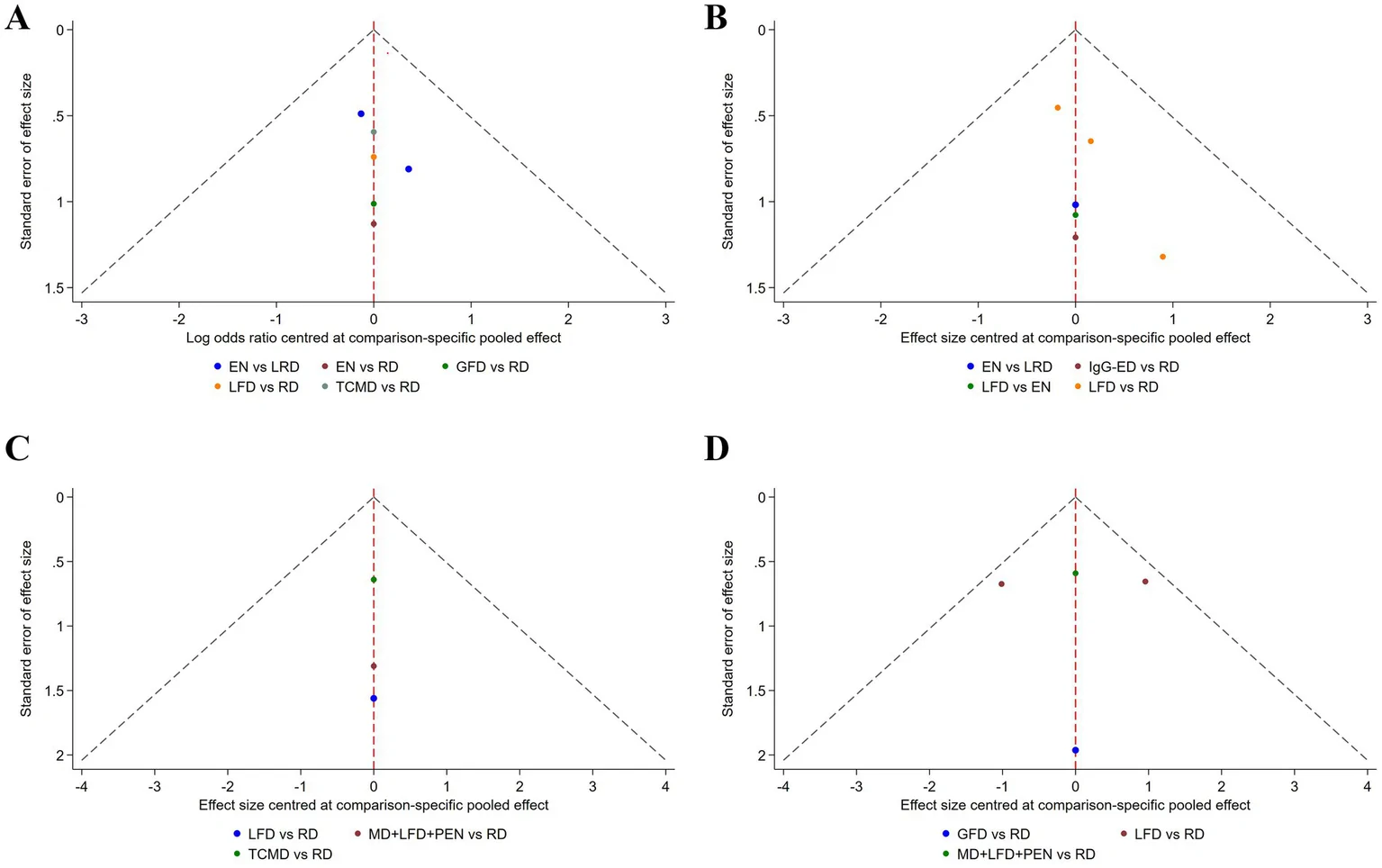
Comparison adjusted funnel plots for the network meta-analyses: **(A)** Clinical effectiveness, **(B)** Albumin, **(C)** Inflammatory Bowel Disease Questionnaire, and **(D)** C-reactive protein. RD, regular diet; MD, Mediterranean diet; LRD, low residue diet; EN, enteral nutrition; PEN, partial enteral nutrition; SCD, specific carbohydrate diet; LFD, low-FODMAP diet; IgG-ED, IgG-guided exclusion diet; GFD, gluten free diet; TCMD, traditional Chinese medicine diet.

Comparison adjusted funnel plots were generated for clinical effectiveness, ALB, IBDQ, and CRP, with no marked asymmetry observed. Egger type tests detected no significant small study effects for clinical effectiveness (intercept = 0.37, *p* = 0.26) or ALB (intercept = 0.56, *p* = 0.16). The test was not estimable for IBDQ because only three studies were available, while no significant small study effect was detected for CRP (intercept = −0.10, *p* = 0.97). As fewer than 10 studies contributed to each outcome, these findings should be interpreted cautiously and do not rule out publication bias (Figure 4).

## 4 Discussion

### 4.1 Principal findings and certainty of evidence

This network meta-analysis included 14 randomized controlled trials evaluating dietary and enteral nutrition interventions in adults with UC. EN was associated with higher odds of clinical effectiveness than LRD, while no other statistically significant differences were observed for this outcome. For ALB, EN, IgG-ED, LFD, and RD were associated with higher levels than LRD. No statistically significant differences were identified for IBDQ or CRP. Although SUCRA rankings placed EN, IgG-ED, MD + LFD + PEN, and GFD in favorable positions for the respective outcomes, these rankings were exploratory and should not be interpreted as evidence of treatment superiority. Overall confidence in the network estimates was low or very low.

### 4.2 Clinical effects of dietary interventions

For clinical effectiveness, EN was associated with higher odds of achieving a favorable clinical outcome than LRD, and this finding remained broadly consistent after excluding studies with a high risk of bias or very small sample sizes. However, the credible intervals remained wide, and the definitions of clinical effectiveness varied across studies, encompassing clinical remission, clinical response, and overall effectiveness, with several studies not reporting explicit assessment criteria. This outcome heterogeneity may have reduced the clinical comparability of the included studies; therefore, the pooled estimate should be interpreted as reflecting overall clinical benefit rather than clinical remission alone. EN may nevertheless offer potential benefits by providing a controlled nutritional composition, reducing exposure to poorly tolerated dietary components, and maintaining adequate energy and nutrient intake during active disease (36–38). Accordingly, EN may represent a promising therapeutic option, but the low confidence in the network estimates precludes its recommendation as a standard approach. Further trials using standardized definitions of clinical response and remission are required.

For ALB, EN, IgG-ED, LFD, and RD were associated with higher levels than LRD, whereas no statistically significant differences were observed among the remaining interventions. IgG-ED had the highest SUCRA value, followed by LFD; however, these rankings were exploratory and should not be interpreted as evidence of superiority. Low albumin levels are commonly associated with active inflammation, increased catabolic activity, and adverse clinical outcomes, while hypoalbuminemia at UC diagnosis may indicate a poorer prognosis (39–41). Nevertheless, albumin is not a specific marker of nutritional status because it may also be affected by systemic inflammation, intestinal protein loss, disease activity, and hydration status (42, 43). Accordingly, the observed increases in ALB should not be interpreted solely as reflecting improved nutritional status. The sparse evidence network and low confidence in the relevant estimates further limit the clinical significance of these findings.

No statistically significant differences were observed among the interventions for IBDQ. Although the combined MD, LFD, and PEN intervention had the highest SUCRA value, only three studies contributed to the network, and the point estimates did not consistently favor this strategy. Its favorable ranking should therefore be regarded as hypothesis generating rather than as evidence of a meaningful improvement in quality of life. The potential benefits of this combined intervention may be related to complementary effects of its individual components: MD may improve overall diet quality, modulate the gut microbiota, and support intestinal homeostasis (11); LFD may alleviate gastrointestinal symptoms by reducing fermentable substrates and luminal osmotic load (10); and PEN may help maintain adequate energy and protein intake (43). However, because these components were evaluated as a multicomponent strategy, their individual contributions could not be determined. Given the limited evidence base and low confidence in the network estimates, further well designed trials are needed to clarify the effects of dietary interventions on quality of life in adults with UC.

For CRP, no statistically significant differences were observed among GFD, LFD, RD, and the combined MD, LFD, and PEN intervention. Although the point estimates numerically favored GFD, which also had the highest SUCRA value, followed by RD, the wide credible intervals and sparse evidence network precluded firm conclusions regarding the comparative effects of these interventions on CRP levels. One possible explanation for this numerical finding is that excluding gluten containing cereals may also reduce exposure to other wheat derived components, including amylase trypsin inhibitors, which have been implicated in innate immune activation (44, 45). Nevertheless, a recent triple blind randomized controlled trial found no significant effects of GFD on CRP or other inflammatory markers in patients with mild to moderate UC, further highlighting the uncertainty surrounding this finding (12). Moreover, CRP is a nonspecific marker of systemic inflammation rather than a direct indicator of intestinal mucosal inflammation and should therefore be interpreted alongside clinical symptoms, other biomarkers, and endoscopic findings (46, 47). Accordingly, the CRP ranking should be regarded as exploratory and should not be interpreted as evidence that GFD is superior to the other dietary strategies.

### 4.3 Strengths and limitations

This study has several strengths. First, the review protocol was prospectively registered, and the study was conducted in accordance with established reporting guidelines for network meta-analysis. Second, only randomized controlled trials were included, thereby reducing the potential for confounding associated with nonrandomized evidence. Third, the analysis focused specifically on adults with UC, enhancing the clinical relevance of the findings for this population. The study also evaluated multiple clinically important outcomes, including clinical effectiveness, nutritional status, systemic inflammation, and health-related quality of life. Methodological rigor was further strengthened by the use of the RoB 2 tool, CINeMA assessment, and sensitivity analyses to evaluate the robustness and certainty of the network estimates.

Several limitations should be acknowledged. First, the evidence base was sparse, with several interventions evaluated in only one or two trials, resulting in wide credible intervals and potentially unstable treatment rankings. Second, the transitivity assumption may have been compromised by differences across studies in disease severity, treatment phase, intervention duration, baseline nutritional status, and concomitant pharmacological therapy. Although background treatment was generally balanced between groups within individual trials, medication details were often incompletely reported, limiting assessment of their influence on treatment effects. Third, none of the outcome networks contained closed loops, precluding formal evaluation of inconsistency between direct and indirect evidence. Finally, confidence in all network estimates was low or very low, and variation in the definitions of clinical effectiveness further reduced the comparability of the pooled results. These limitations should be considered when interpreting the relative effect estimates and SUCRA rankings.

### 4.4 Clinical implications and future research

Given the low or very low confidence in the available evidence, dietary and enteral nutrition interventions should be considered individualized adjunctive strategies rather than substitutes for standard pharmacological treatment. The selection of an appropriate intervention should take into account disease severity, treatment phase, nutritional status, symptom profile, patient preferences, and the feasibility of long term adherence.

Future trials should adopt standardized definitions of clinical remission and response and clearly describe the composition, dose, duration, and delivery of each intervention. Background pharmacological therapy should be applied consistently across study groups and reported in sufficient detail, while adherence and intervention fidelity should be systematically assessed. In addition to clinical symptoms and laboratory measures, future studies should evaluate endoscopic remission, fecal calprotectin, safety, relapse, and long-term outcomes. Adequately powered multicenter trials should also report outcomes stratified by disease severity and treatment phase to improve the precision of future research.

## 5 Conclusion

Current evidence suggests that EN may be associated with greater clinical effectiveness than LRD, while several dietary and enteral nutrition interventions may improve ALB levels compared with LRD. Further large and rigorously designed randomized controlled trials are needed to provide more robust evidence for dietary interventions in adults with UC.

## Data Availability

The original contributions presented in the study are included in the article/Supplementary material, further inquiries can be directed to the corresponding authors.
