# Supplementary material for: Comparative efficacy of dietary interventions and enteral nutrition in adults with ulcerative colitis: a network meta-analysis
Source: Front Nutr. 2026 Aug 25;13:1886706. doi: 10.3389/fnut.2026.1886706 (PMC13549872; doi:10.3389/fnut.2026.1886706)
Supplement: Supplementary file 1 [file Data_Sheet_1.PDF]

Confidence In Network Meta Analysis - CINeMA 2.0.0 - Project: **有效性_CINeMA_ready_upload_all_M**

有效性_CINeMA_ready_upload_all_M

Comparison	Number of Studies	Within-study bias	Reporting bias	Indirectness	Imprecision	Heterogeneity	Incoherence	Confidence rating	Reason(s) for downgrading
Mixed evidence									
EN vs LRD	2	Some concerns ☑<br>**Majority RoB:** Some concerns<br>**Average RoB:** Some concerns (applied)<br>**Highest RoB:** Some concerns	Some concerns ☑<br>**Evidence:** mixed<br>**Source:** manual	Some concerns ☑<br>**Majority:** moderate<br>**Average:** moderate<br>**Highest:** moderate	No concerns<br>**NMA effect:** 4.368<br>**95% CI:** (1.924, 9.919)<br>crosses 0 clinically important line(s)<br>**Clinically important:** (0.6666666666666666, 1.5)	Some concerns ☑<br>**NMA effect:** 4.368<br>**95% CI:** (1.924, 9.919)<br>**95% PrI:** (0.722, 26.435)<br>**τ^2:** 0.000<br>**I^2:** 0.0%%	No concerns<br>**Evidence:** direct only<br>IF not applicable; judged from the design-by-treatment test.	Low	| Within-study bias | Reporting bias | Indirectness | Heterogeneity |

Comparison	Number of Studies	Within-study bias	Reporting bias	Indirectness	Imprecision	Heterogeneity	Incoherence	Confidence rating	Reason(s) for downgrading
**EN vs RD**	1	Some concerns ☑<br>**Majority RoB:** Some concerns<br>**Average RoB:** Some concerns (applied)<br>**Highest RoB:** Some concerns	Some concerns ☑<br>**Evidence:** mixed<br>**Source:** manual	Some concerns ☑<br>**Majority:** moderate<br>**Average:** moderate<br>**Highest:** moderate	Some concerns ☑<br>**NMA effect:** 8.250<br>**95% CI:** (0.902, 75.415)<br>crosses 1 clinically important line(s)<br>**Clinically important:** (0.66666666666 66666, 1.5)	Some concerns ☑<br>**NMA effect:** 8.250<br>**95% CI:** (0.902, 75.415)<br>**95% PrI:** (0.064, 1061.990)<br>**τ^2:** —<br>**I^2:** null%	No concerns<br>**Evidence:** direct only<br>IF not applicable; judged from the design-by-treatment test.	Very low	| Within-study bias | Reporting bias | Indirectness | Imprecision | Heterogeneity |

Comparison	Number of Studies	Within-study bias	Reporting bias	Indirectness	Imprecision	Heterogeneity	Incoherence	Confidence rating	Reason(s) for downgrading
**GFD vs RD**	1	Some concerns ☑<br>**Majority RoB:** Some concerns<br>**Average RoB:** Some concerns (applied)<br>**Highest RoB:** Some concerns	Some concerns ☑<br>**Evidence:** mixed<br>**Source:** manual	Some concerns ☑<br>**Majority:** moderate<br>**Average:** moderate<br>**Highest:** moderate	Major concerns ☑<br>**NMA effect:** 1.650<br>**95% CI:** (0.227, 11.994)<br>crosses 2 clinically important line(s)<br>**Clinically important:** (0.6666666666666666, 1.5)	No concerns<br>**NMA effect:** 1.650<br>**95% CI:** (0.227, 11.994)<br>**95% PrI:** (0.021, 128.419)<br>**τ^2:** —<br>**I^2:** null%	No concerns<br>**Evidence:** direct only<br>IF not applicable; judged from the design-by-treatment test.	Very low	| Within-study bias | Reporting bias | Indirectness | Imprecision |

Comparison	Number of Studies	Within-study bias	Reporting bias	Indirectness	Imprecision	Heterogeneity	Incoherence	Confidence rating	Reason(s) for downgrading
**LFD vs RD**	2	Some concerns ☑ **Majority RoB:** Some concerns **Average RoB:** Some concerns (applied) **Highest RoB:** Some concerns	Some concerns ☑ **Evidence:** mixed **Source:** manual	Some concerns ☑ **Majority:** moderate **Average:** moderate **Highest:** moderate	No concerns **NMA effect:** 2.987 **95% CI:** (1.071, 8.328) crosses 0 clinically important line(s) **Clinically important:** (0.6666666666666666, 1.5)	Major concerns ☑ **NMA effect:** 2.987 **95% CI:** (1.071, 8.328) **95% PrI:** (0.314, 28.366) **τ^2:** 0.000 **I^2:** 0.0%%	No concerns **Evidence:** direct only IF not applicable; judged from the design-by-treatment test.	Very low	| Within-study bias | Reporting bias | Indirectness | Heterogeneity |

Comparison	Number of Studies	Within-study bias	Reporting bias	Indirectness	Imprecision	Heterogeneity	Incoherence	Confidence rating	Reason(s) for downgrading
**RD vs TCMD**	1	Some concerns ☑<br>**Majority RoB:** Some concerns<br>**Average RoB:** Some concerns (applied)<br>**Highest RoB:** Some concerns	Some concerns ☑<br>**Evidence:** mixed<br>**Source:** manual	Some concerns ☑<br>**Majority:** moderate<br>**Average:** moderate<br>**Highest:** moderate	Major concerns ☑<br>**NMA effect:** 0.770<br>**95% CI:** (0.240, 2.468)<br>crosses 2 clinically important line(s)<br>**Clinically important:** (0.6666666666666666, 1.5)	No concerns<br>**NMA effect:** 0.770<br>**95% CI:** (0.240, 2.468)<br>**95% PrI:** (0.060, 9.935)<br>**τ^2:** —<br>**I^2:** null%	No concerns<br>**Evidence:** direct only<br>IF not applicable; judged from the design-by-treatment test.	Very low	| Within-study bias | Reporting bias | Indirectness | Imprecision |

Indirect evidence

Comparison	Number of Studies	Within-study bias	Reporting bias	Indirectness	Imprecision	Heterogeneity	Incoherence	Confidence rating	Reason(s) for downgrading
EN vs GFD	--	Some concerns ☑<br>**Majority RoB:** Some concerns<br>**Average RoB:** Some concerns (applied)<br>**Highest RoB:** Some concerns	Some concerns ☑<br>**Evidence:** mixed<br>**Source:** manual	Some concerns ☑	Major concerns ☑<br>**NMA effect:** 5.000<br>**95% CI:** (0.256, 97.622)<br>crosses 2 clinically important line(s)<br>**Clinically important:** (0.6666666666666666, 1.5)	No concerns<br>**NMA effect:** 5.000<br>**95% CI:** (0.256, 97.622)<br>**95% PrI:** (0.007, 3405.340)	No concerns<br>**Evidence:** indirect only<br>IF not applicable; judged from the design-by-treatment test.	Very low	| Within-study bias | Reporting bias | Indirectness | Imprecision |

Comparison	Number of Studies	Within-study bias	Reporting bias	Indirectness	Imprecision	Heterogeneity	Incoherence	Confidence rating	Reason(s) for downgrading
**EN vs LFD**	--	Some concerns ☑<br>**Majority RoB:** Some concerns<br>**Average RoB:** Some concerns (applied)<br>**Highest RoB:** Some concerns	Some concerns ☑<br>**Evidence:** mixed<br>**Source:** manual	Some concerns ☑	Major concerns ☑<br>**NMA effect:** 2.762<br>**95% CI:** (0.241, 31.655)<br>crosses 2 clinically important line(s)<br>**Clinically important:** (0.6666666666666666, 1.5)	No concerns<br>**NMA effect:** 2.762<br>**95% CI:** (0.241, 31.655)<br>**95% PrI:** (0.013, 583.999)	No concerns<br>**Evidence:** indirect only<br>IF not applicable; judged from the design-by-treatment test.	Very low	| Within-study bias | Reporting bias | Indirectness | Imprecision |

<table>
<tr><th>Comparison</th><th>Number of Studies</th><th>Within-study bias</th><th>Reporting bias</th><th>Indirectness</th><th>Imprecision</th><th>Heterogeneity</th><th>Incoherence</th><th>Confidence rating</th><th>Reason(s) for downgrading</th></tr>
<tr><td>EN vs TCMD</td><td>--</td><td>Some concerns ☑
Majority RoB: Some concerns
Average RoB: Some concerns (applied)
Highest RoB: Some concerns</td><td>Some concerns ☑
Evidence: mixed
Source: manual</td><td>Some concerns ☑</td><td>Major concerns ☑
NMA effect: 6.352
95% CI: (0.521, 77.447)
crosses 2 clinically important line(s)
Clinically important: (0.6666666666666666, 1.5)</td><td>No concerns
NMA effect: 6.352
95% CI: (0.521, 77.447)
95% PrI: (0.026, 1538.557)</td><td>No concerns
Evidence: indirect only
IF not applicable; judged from the design-by-treatment test.</td><td>Very low</td><td>| Within-study bias | Reporting bias | Indirectness | Imprecision |</td></tr>
</table>

Comparison	Number of Studies	Within-study bias	Reporting bias	Indirectness	Imprecision	Heterogeneity	Incoherence	Confidence rating	Reason(s) for downgrading
**GFD vs LFD**	--	Some concerns ☑<br>**Majority RoB:** Some concerns<br>**Average RoB:** Some concerns (applied)<br>**Highest RoB:** Some concerns	Some concerns ☑<br>**Evidence:** mixed<br>**Source:** manual	Some concerns ☑	Major concerns ☑<br>**NMA effect:** 0.552<br>**95% CI:** (0.059, 5.153)<br>crosses 2 clinically important line(s)<br>**Clinically important:** (0.6666666666666666, 1.5)	No concerns<br>**NMA effect:** 0.552<br>**95% CI:** (0.059, 5.153)<br>**95% PrI:** (0.004, 74.336)	No concerns<br>**Evidence:** indirect only<br>IF not applicable; judged from the design-by-treatment test.	Very low	| Within-study bias | Reporting bias | Indirectness | Imprecision |

Comparison	Number of Studies	Within-study bias	Reporting bias	Indirectness	Imprecision	Heterogeneity	Incoherence	Confidence rating	Reason(s) for downgrading
**GFD vs LRD**	--	Some concerns ☑<br>**Majority RoB:** Some concerns<br>**Average RoB:** Some concerns (applied)<br>**Highest RoB:** Some concerns	Some concerns ☑<br>**Evidence:** mixed<br>**Source:** manual	Some concerns ☑	Major concerns ☑<br>**NMA effect:** 0.874<br>**95% CI:** (0.040, 19.062)<br>crosses 2 clinically important line(s)<br>**Clinically important:** (0.6666666666666666, 1.5)	No concerns<br>**NMA effect:** 0.874<br>**95% CI:** (0.040, 19.062)<br>**95% PrI:** (0.001, 759.378)	No concerns<br>**Evidence:** indirect only<br>IF not applicable; judged from the design-by-treatment test.	Very low	| Within-study bias | Reporting bias | Indirectness | Imprecision |

Comparison	Number of Studies	Within-study bias	Reporting bias	Indirectness	Imprecision	Heterogeneity	Incoherence	Confidence rating	Reason(s) for downgrading
GFD vs TCMD	--	Some concerns ☑<br>**Majority RoB:** Some concerns<br>**Average RoB:** Some concerns (applied)<br>**Highest RoB:** Some concerns	Some concerns ☑<br>**Evidence:** mixed<br>**Source:** manual	Some concerns ☑	Major concerns ☑<br>**NMA effect:** 1.270<br>**95% CI:** (0.127, 12.677)<br>crosses 2 clinically important line(s)<br>**Clinically important:** (0.6666666666666666, 1.5)	No concerns<br>**NMA effect:** 1.270<br>**95% CI:** (0.127, 12.677)<br>**95% PrI:** (0.008, 198.224)	No concerns<br>**Evidence:** indirect only<br>IF not applicable; judged from the design-by-treatment test.	Very low	| Within-study bias | Reporting bias | Indirectness | Imprecision |

Comparison	Number of Studies	Within-study bias	Reporting bias	Indirectness	Imprecision	Heterogeneity	Incoherence	Confidence rating	Reason(s) for downgrading
**LFD vs LRD**	--	Some concerns ☑ **Majority RoB:** Some concerns **Average RoB:** Some concerns (applied) **Highest RoB:** Some concerns	Some concerns ☑ **Evidence:** mixed **Source:** manual	Some concerns ☑	Major concerns ☑ **NMA effect:** 1.581 **95% CI:** (0.121, 20.726) crosses 2 clinically important line(s) **Clinically important:** (0.6666666666666666, 1.5)	No concerns **NMA effect:** 1.581 **95% CI:** (0.121, 20.726) **95% PrI:** (0.006, 448.900)	No concerns **Evidence:** indirect only IF not applicable; judged from the design-by-treatment test.	Very low	| Within-study bias | Reporting bias | Indirectness | Imprecision |

Comparison	Number of Studies	Within-study bias	Reporting bias	Indirectness	Imprecision	Heterogeneity	Incoherence	Confidence rating	Reason(s) for downgrading
LFD vs TCMD	--	Some concerns ☑ **Majority RoB:** Some concerns **Average RoB:** Some concerns (applied) **Highest RoB:** Some concerns	Some concerns ☑ **Evidence:** mixed **Source:** manual	Some concerns ☑	Major concerns ☑ **NMA effect:** 2.300 **95% CI:** (0.487, 10.857) crosses 2 clinically important line(s) **Clinically important:** (0.6666666666666666, 1.5)	No concerns **NMA effect:** 2.300 **95% CI:** (0.487, 10.857) **95% PrI:** (0.076, 69.401)	No concerns **Evidence:** indirect only IF not applicable; judged from the design-by-treatment test.	Very low	| Within-study bias | Reporting bias | Indirectness | Imprecision |

Comparison	Number of Studies	Within-study bias	Reporting bias	Indirectness	Imprecision	Heterogeneity	Incoherence	Confidence rating	Reason(s) for downgrading
**LRD vs RD**	--	Some concerns ☑<br>**Majority RoB:** Some concerns<br>**Average RoB:** Some concerns (applied)<br>**Highest RoB:** Some concerns	Some concerns ☑<br>**Evidence:** mixed<br>**Source:** manual	Some concerns ☑	Major concerns ☑<br>**NMA effect:** 1.889<br>**95% CI:** (0.178, 19.999)<br>crosses 2 clinically important line(s)<br>**Clinically important:** (0.6666666666666666, 1.5)	No concerns<br>**NMA effect:** 1.889<br>**95% CI:** (0.178, 19.999)<br>**95% PrI:** (0.011, 335.761)	No concerns<br>**Evidence:** indirect only<br>IF not applicable; judged from the design-by-treatment test.	Very low	| Within-study bias | Reporting bias | Indirectness | Imprecision |

Comparison	Number of Studies	Within-study bias	Reporting bias	Indirectness	Imprecision	Heterogeneity	Incoherence	Confidence rating	Reason(s) for downgrading
LRD vs TCMD	--	Some concerns ☑ **Majority RoB:** Some concerns **Average RoB:** Some concerns (applied) **Highest RoB:** Some concerns	Some concerns ☑ **Evidence:** mixed **Source:** manual	Some concerns ☑	Major concerns ☑ **NMA effect:** 1.454 **95% CI:** (0.105, 20.210) crosses 2 clinically important line(s) **Clinically important:** (0.6666666666666666, 1.5)	No concerns **NMA effect:** 1.454 **95% CI:** (0.105, 20.210) **95% PrI:** (0.005, 469.609)	No concerns **Evidence:** indirect only IF not applicable; judged from the design-by-treatment test.	Very low	| Within-study bias | Reporting bias | Indirectness | Imprecision |

Confidence In Network Meta Analysis - CINeMA 2.0.0 - Project: **ALB_CINeMA_ready_upload_all_M**

ALB_CINeMA_ready_upload_all_M

Comparison	Number of Studies	Within-study bias	Reporting bias	Indirectness	Imprecision	Heterogeneity	Incoherence	Confidence rating	Reason(s) for downgrading
Mixed evidence									
**EN vs LFD**	1	Some concerns ☑ **Majority RoB:** Some concerns **Average RoB:** Some concerns (applied) **Highest RoB:** Some concerns	Some concerns ☑ **Evidence:** mixed **Source:** manual	Some concerns ☑ **Majority:** moderate **Average:** moderate **Highest:** moderate	No concerns **NMA effect:** -3.140 **95% CI:** (-5.252, -1.028) crosses 0 clinically important line(s) **Clinically important:** (-3, 3)	Some concerns ☑ **NMA effect:** -3.140 **95% CI:** (-5.252, -1.028) **95% PrI:** (-7.776, 1.496) **τ^2:** — **I^2:** null%	No concerns **Evidence:** direct only IF not applicable; judged from the design-by-treatment test.	Low	| Within-study bias | Reporting bias | Indirectness | Heterogeneity |

Comparison	Number of Studies	Within-study bias	Reporting bias	Indirectness	Imprecision	Heterogeneity	Incoherence	Confidence rating	Reason(s) for downgrading
EN vs LRD	1	Some concerns ☑ **Majority RoB:** Some concerns **Average RoB:** Some concerns (applied) **Highest RoB:** Some concerns	Some concerns ☑ **Evidence:** mixed **Source:** manual	Some concerns ☑ **Majority:** moderate **Average:** moderate **Highest:** moderate	No concerns **NMA effect:** 4.120 **95% CI:** (2.125, 6.115) crosses 0 clinically important line(s) **Clinically important:** (-3, 3)	Some concerns ☑ **NMA effect:** 4.120 **95% CI:** (2.125, 6.115) **95% PrI:** (-0.260, 8.500) **τ^2:** — **I^2:** null%	No concerns **Evidence:** direct only IF not applicable; judged from the design-by-treatment test.	Low	| Within-study bias | Reporting bias | Indirectness | Heterogeneity |

Comparison	Number of Studies	Within-study bias	Reporting bias	Indirectness	Imprecision	Heterogeneity	Incoherence	Confidence rating	Reason(s) for downgrading
**IgG-ED vs RD**	1	Some concerns ☑<br>**Majority RoB:** Some concerns<br>**Average RoB:** Some concerns (applied)<br>**Highest RoB:** Some concerns	Some concerns ☑<br>**Evidence:** mixed<br>**Source:** manual	Some concerns ☑<br>**Majority:** moderate<br>**Average:** moderate<br>**Highest:** moderate	Some concerns ☑<br>**NMA effect:** 2.330<br>**95% CI:** (-0.037, 4.697)<br>crosses 1 clinically important line(s)<br>**Clinically important:** (-3, 3)	No concerns<br>**NMA effect:** 2.330<br>**95% CI:** (-0.037, 4.697)<br>**95% PrI:** (-2.867, 7.527)<br>**τ^2:** —<br>**I^2:** null%	No concerns<br>**Evidence:** direct only<br>IF not applicable; judged from the design-by-treatment test.	Low	| Within-study bias | Reporting bias | Indirectness | Imprecision |

Comparison	Number of Studies	Within-study bias	Reporting bias	Indirectness	Imprecision	Heterogeneity	Incoherence	Confidence rating	Reason(s) for downgrading
**LFD vs RD**	3	Some concerns ☑<br>**Majority RoB:** Some concerns<br>**Average RoB:** Some concerns (applied)<br>**Highest RoB:** Some concerns	Some concerns ☑<br>**Evidence:** mixed<br>**Source:** manual	Some concerns ☑<br>**Majority:** moderate<br>**Average:** moderate<br>**Highest:** moderate	No concerns<br>**NMA effect:** 0.633<br>**95% CI:** (-0.068, 1.334) crosses 0 clinically important line(s)<br>**Clinically important:** (-3, 3)	No concerns<br>**NMA effect:** 0.633<br>**95% CI:** (-0.068, 1.334)<br>**95% PrI:** (-0.906, 2.172)<br>**τ^2:** 0.000<br>**I^2:** 0.0%%	No concerns<br>**Evidence:** direct only<br>IF not applicable; judged from the design-by-treatment test.	Low	| Within-study bias | Reporting bias | Indirectness |

Indirect evidence

Comparison	Number of Studies	Within-study bias	Reporting bias	Indirectness	Imprecision	Heterogeneity	Incoherence	Confidence rating	Reason(s) for downgrading
**EN vs IgG-ED**	--	Some concerns ☑ **Majority RoB:** Some concerns **Average RoB:** Some concerns (applied) **Highest RoB:** Some concerns	Some concerns☑ **Evidence:** mixed **Source:** manual	Some concerns☑	No concerns **NMA effect:** -4.837 **95% CI:** (-8.086, -1.588) crosses 0 clinically important line(s) **Clinically important:** (-3, 3)	Some concerns☑ **NMA effect:** -4.837 **95% CI:** (-8.086, -1.588) **95% PrI:** (-11.969, 2.295)	No concerns **Evidence:** indirect only IF not applicable; judged from the design-by-treatment test.	Low	| Within-study bias | Reporting bias | Indirectness | Heterogeneity |

Comparison	Number of Studies	Within-study bias	Reporting bias	Indirectness	Imprecision	Heterogeneity	Incoherence	Confidence rating	Reason(s) for downgrading
**EN vs RD**	--	Some concerns ☑<br>**Majority RoB:** Some concerns<br>**Average RoB:** Some concerns (applied)<br>**Highest RoB:** Some concerns	Some concerns ☑<br>**Evidence:** mixed<br>**Source:** manual	Some concerns ☑	No concerns<br>**NMA effect:** -2.507<br>**95% CI:** (-4.732, -0.282)<br>crosses 0 clinically important line(s)<br>**Clinically important:** (-3, 3)	Some concerns ☑<br>**NMA effect:** -2.507<br>**95% CI:** (-4.732, -0.282)<br>**95% PrI:** (-7.391, 2.377)	No concerns<br>**Evidence:** indirect only<br>IF not applicable; judged from the design-by-treatment test.	Low	| Within-study bias | Reporting bias | Indirectness | Heterogeneity |

Comparison	Number of Studies	Within-study bias	Reporting bias	Indirectness	Imprecision	Heterogeneity	Incoherence	Confidence rating	Reason(s) for downgrading
IgG-ED vs LFD	--	Some concerns ☑<br>**Majority RoB:** Some concerns<br>**Average RoB:** Some concerns (applied)<br>**Highest RoB:** Some concerns	Some concerns ☑<br>**Evidence:** mixed<br>**Source:** manual	Some concerns ☑	Some concerns ☑<br>**NMA effect:** 1.697<br>**95% CI:** (-0.772, 4.166)<br>crosses 1 clinically important line(s)<br>**Clinically important:** (-3, 3)	Some concerns ☑<br>**NMA effect:** 1.697<br>**95% CI:** (-0.772, 4.166)<br>**95% PrI:** (-3.723, 7.117)	No concerns<br>**Evidence:** indirect only<br>IF not applicable; judged from the design-by-treatment test.	Very low	| Within-study bias | Reporting bias | Indirectness | Imprecision | Heterogeneity |

Comparison	Number of Studies	Within-study bias	Reporting bias	Indirectness	Imprecision	Heterogeneity	Incoherence	Confidence rating	Reason(s) for downgrading
**IgG-ED vs LRD**	--	Some concerns ☑ **Majority RoB:** Some concerns **Average RoB:** Some concerns (applied) **Highest RoB:** Some concerns	Some concerns ☑ **Evidence:** mixed **Source:** manual	Some concerns ☑	No concerns **NMA effect:** 8.957 **95% CI:** (5.145, 12.770) crosses 0 clinically important line(s) **Clinically important:** (-3, 3)	No concerns **NMA effect:** 8.957 **95% CI:** (5.145, 12.770) **95% PrI:** (0.588, 17.327)	No concerns **Evidence:** indirect only IF not applicable; judged from the design-by-treatment test.	Low	| Within-study bias | Reporting bias | Indirectness |

Comparison	Number of Studies	Within-study bias	Reporting bias	Indirectness	Imprecision	Heterogeneity	Incoherence	Confidence rating	Reason(s) for downgrading
**LFD vs LRD**	--	Some concerns ☑<br>**Majority RoB:** Some concerns<br>**Average RoB:** Some concerns (applied)<br>**Highest RoB:** Some concerns	Some concerns ☑<br>**Evidence:** mixed<br>**Source:** manual	Some concerns ☑	No concerns<br>**NMA effect:** 7.260<br>**95% CI:** (4.355, 10.165)<br>crosses 0 clinically important line(s)<br>**Clinically important:** (-3, 3)	No concerns<br>**NMA effect:** 7.260<br>**95% CI:** (4.355, 10.165)<br>**95% PrI:** (0.882, 13.637)	No concerns<br>**Evidence:** indirect only<br>IF not applicable; judged from the design-by-treatment test.	Low	| Within-study bias | Reporting bias | Indirectness |

Comparison	Number of Studies	Within-study bias	Reporting bias	Indirectness	Imprecision	Heterogeneity	Incoherence	Confidence rating	Reason(s) for downgrading
**LRD vs RD**	--	Some concerns ☑<br>**Majority RoB:** Some concerns<br>**Average RoB:** Some concerns (applied)<br>**Highest RoB:** Some concerns	Some concerns☑<br>**Evidence:** mixed<br>**Source:** manual	Some concerns☑	No concerns<br>**NMA effect:** -6.627<br>**95% CI:** (-9.616, -3.639)<br>crosses 0 clinically important line(s)<br>**Clinically important:** (-3, 3)	No concerns<br>**NMA effect:** -6.627<br>**95% CI:** (-9.616, -3.639)<br>**95% PrI:** (-13.188, -0.067)	No concerns<br>**Evidence:** indirect only<br>IF not applicable; judged from the design-by-treatment test.	Low	| Within-study bias | Reporting bias | Indirectness |

Confidence In Network Meta Analysis - CINeMA 2.0.0 - Project: **IBDQ_CINeMA_ready_upload_all_M (1)**

IBDQ_CINeMA_ready_upload_all_M (1)

Comparison	Number of Studies	Within-study bias	Reporting bias	Indirectness	Imprecision	Heterogeneity	Incoherence	Confidence rating	Reason(s) for downgrading
Mixed evidence									
**LFD vs RD**	1	Some concerns ☑ **Majority RoB:** — **Average RoB:** (applied) **Highest RoB:** Some concerns	Some concerns ☑ **Evidence:** mixed **Source:** manual	Some concerns ☑ **Majority:** moderate **Average:** moderate **Highest:** moderate	No concerns **NMA effect:** 9.370 **95% CI:** (6.311, 12.429) crosses 0 clinically important line(s) **Clinically important:** (-16, 16)	No concerns **NMA effect:** 9.370 **95% CI:** (6.311, 12.429) **95% PrI:** (6.311, 12.429) **τ^2:** — **I^2:** null%	No concerns **Evidence:** direct only IF not applicable; judged from the design-by-treatment test.	Low	| Within-study bias | Reporting bias | Indirectness |

Comparison	Number of Studies	Within-study bias	Reporting bias	Indirectness	Imprecision	Heterogeneity	Incoherence	Confidence rating	Reason(s) for downgrading
MD+LFD+PEN vs RD	1	Some concerns ☑ **Majority RoB:** — **Average RoB:** (applied) **Highest RoB:** Some concerns	Some concerns ☑ **Evidence:** mixed **Source:** manual	Some concerns☑ **Majority:** moderate **Average:** moderate **Highest:** moderate	No concerns **NMA effect:** 12.500 **95% CI:** (9.931, 15.069) crosses 0 clinically important line(s) **Clinically important:** (-16, 16)	No concerns **NMA effect:** 12.500 **95% CI:** (9.931, 15.069) **95% PrI:** (9.931, 15.069) **τ^2:** — **I^2:** null%	No concerns **Evidence:** direct only IF not applicable; judged from the design-by-treatment test.	Low	| Within-study bias | Reporting bias | Indirectness |
RD vs TCMD	1	Some concerns ☑ **Majority RoB:** — **Average RoB:** (applied) **Highest RoB:** Some concerns	Some concerns ☑ **Evidence:** mixed **Source:** manual	Some concerns☑ **Majority:** moderate **Average:** moderate **Highest:** moderate	No concerns **NMA effect:** -1.340 **95% CI:** (-2.594, -0.086) crosses 0 clinically important line(s) **Clinically important:** (-16, 16)	No concerns **NMA effect:** -1.340 **95% CI:** (-2.594, -0.086) **95% PrI:** (-2.594, -0.086) **τ^2:** — **I^2:** null%	No concerns **Evidence:** direct only IF not applicable; judged from the design-by-treatment test.	Low	| Within-study bias | Reporting bias | Indirectness |

Indirect evidence

Comparison	Number of Studies	Within-study bias	Reporting bias	Indirectness	Imprecision	Heterogeneity	Incoherence	Confidence rating	Reason(s) for downgrading
**LFD vs MD+LFD+PEN**	--	Some concerns ☑<br>**Majority RoB:** —<br>**Average RoB:** (applied)<br>**Highest RoB:** Some concerns	Some concerns ☑<br>**Evidence:** mixed<br>**Source:** manual	Some concerns☑	No concerns<br>**NMA effect:** -3.130<br>**95% CI:** (-7.124, 0.864)<br>crosses 0 clinically important line(s)<br>**Clinically important:** (-16, 16)	No concerns<br>**NMA effect:** -3.130<br>**95% CI:** (-7.124, 0.864)<br>**95% PrI:** (-7.124, 0.864)	No concerns<br>**Evidence:** indirect only<br>IF not applicable; judged from the design-by-treatment test.	Low	| Within-study bias | Reporting bias | Indirectness |
**LFD vs TCMD**	--	Some concerns ☑<br>**Majority RoB:** —<br>**Average RoB:** (applied)<br>**Highest RoB:** Some concerns	Some concerns ☑<br>**Evidence:** mixed<br>**Source:** manual	Some concerns☑	No concerns<br>**NMA effect:** 8.030<br>**95% CI:** (4.724, 11.336)<br>crosses 0 clinically important line(s)<br>**Clinically important:** (-16, 16)	No concerns<br>**NMA effect:** 8.030<br>**95% CI:** (4.724, 11.336)<br>**95% PrI:** (4.724, 11.336)	No concerns<br>**Evidence:** indirect only<br>IF not applicable; judged from the design-by-treatment test.	Low	| Within-study bias | Reporting bias | Indirectness |

Comparison	Number of Studies	Within-study bias	Reporting bias	Indirectness	Imprecision	Heterogeneity	Incoherence	Confidence rating	Reason(s) for downgrading
**MD+LFD+PEN vs TCMD**	--	Some concerns ☑<br>**Majority RoB:** —<br>**Average RoB:** (applied)<br>**Highest RoB:** Some concerns	Some concerns ☑<br>**Evidence:** mixed<br>**Source:** manual	Some concerns☑	No concerns<br>**NMA effect:** 11.160<br>**95% CI:** (8.302, 14.018)<br>crosses 0 clinically important line(s)<br>**Clinically important:** (-16, 16)	No concerns<br>**NMA effect:** 11.160<br>**95% CI:** (8.302, 14.018)<br>**95% PrI:** (8.302, 14.018)	No concerns<br>**Evidence:** indirect only<br>IF not applicable; judged from the design-by-treatment test.	Low	| Within-study bias | Reporting bias | Indirectness |

Confidence In Network Meta Analysis - CINeMA 2.0.0 - Project: **CRP_CINeMA_ready_upload_all_M(1)**

CRP_CINeMA_ready_upload_all_M(1)

Comparison	Number of Studies	Within-study bias	Reporting bias	Indirectness	Imprecision	Heterogeneity	Incoherence	Confidence rating	Reason(s) for downgrading
Mixed evidence									
GFD vs RD	1	Some concerns<br>**Majority RoB:** Some concerns<br>**Average RoB:** Some concerns (applied)<br>**Highest RoB:** Some concerns	Some concerns<br>**Evidence:** mixed<br>**Source:** manual	Some concerns<br>**Majority:** moderate<br>**Average:** moderate<br>**Highest:** moderate	Major concerns<br>**NMA effect:** 1.050<br>**95% CI:** (-3.484, 5.584)<br>crosses 2 clinically important line(s)<br>**Clinically important:** (-3, 3)	No concerns<br>**NMA effect:** 1.050<br>**95% CI:** (-3.484, 5.584)<br>**95% PrI:** (-3.484, 5.584)<br>**τ^2:** —<br>**I^2:** null%	Some concerns<br>**Evidence:** direct only<br>IF not applicable; judged from the design-by-treatment test.	Very low	| Within-study bias | Reporting bias | Indirectness | Imprecision | Incoherence |

Comparison	Number of Studies	Within-study bias	Reporting bias	Indirectness	Imprecision	Heterogeneity	Incoherence	Confidence rating	Reason(s) for downgrading
**LFD vs RD**	2	Some concerns ☑<br>**Majority RoB:** Some concerns<br>**Average RoB:** Some concerns (applied)<br>**Highest RoB:** Some concerns	Some concerns ☑<br>**Evidence:** mixed<br>**Source:** manual	Some concerns ☑<br>**Majority:** moderate<br>**Average:** moderate<br>**Highest:** moderate	Some concerns ☑<br>**NMA effect:** -1.329<br>**95% CI:** (-3.259, 0.602)<br>crosses 1 clinically important line(s)<br>**Clinically important:** (-3, 3)	No concerns<br>**NMA effect:** -1.329<br>**95% CI:** (-3.259, 0.602)<br>**95% PrI:** (-3.259, 0.602)<br>**τ^2:** 1.499<br>**I^2:** 77.2%%	Some concerns ☑<br>**Evidence:** direct only<br>IF not applicable; judged from the design-by-treatment test.	Very low	| Within-study bias | Reporting bias | Indirectness | Imprecision | Incoherence |

Comparison	Number of Studies	Within-study bias	Reporting bias	Indirectness	Imprecision	Heterogeneity	Incoherence	Confidence rating	Reason(s) for downgrading
**MD+LFD+PEN vs RD**	1	Some concerns ☑<br>**Majority RoB:** Some concerns<br>**Average RoB:** Some concerns (applied)<br>**Highest RoB:** Some concerns	Some concerns ☑<br>**Evidence:** mixed<br>**Source:** manual	Some concerns ☑<br>**Majority:** moderate<br>**Average:** moderate<br>**Highest:** moderate	Some concerns ☑<br>**NMA effect:** -0.950<br>**95% CI:** (-3.614, 1.714)<br>crosses 1 clinically important line(s)<br>**Clinically important:** (-3, 3)	No concerns<br>**NMA effect:** -0.950<br>**95% CI:** (-3.614, 1.714)<br>**95% PrI:** (-3.614, 1.714)<br>**τ^2:** —<br>**I^2:** null%	Some concerns ☑<br>**Evidence:** direct only<br>IF not applicable; judged from the design-by-treatment test.	Very low	| Within-study bias | Reporting bias | Indirectness | Imprecision | Incoherence |

Indirect evidence

Comparison	Number of Studies	Within-study bias	Reporting bias	Indirectness	Imprecision	Heterogeneity	Incoherence	Confidence rating	Reason(s) for downgrading
**GFD vs LFD**	--	Some concerns ☑ **Majority RoB:** Some concerns **Average RoB:** Some concerns (applied) **Highest RoB:** Some concerns	Some concerns☑ **Evidence:** mixed **Source:** manual	Some concerns☑	Some concerns☑ **NMA effect:** 2.378 **95% CI:** (-2.549, 7.306) crosses 1 clinically important line(s) **Clinically important:** (-3, 3)	No concerns **NMA effect:** 2.378 **95% CI:** (-2.549, 7.306) **95% PrI:** (-2.549, 7.306)	Some concerns☑ **Evidence:** indirect only IF not applicable; judged from the design-by-treatment test.	Very low	| Within-study bias | Reporting bias | Indirectness | Imprecision | Incoherence |

Comparison	Number of Studies	Within-study bias	Reporting bias	Indirectness	Imprecision	Heterogeneity	Incoherence	Confidence rating	Reason(s) for downgrading
GFD vs MD+LFD+PEN	--	Some concerns ✓<br>**Majority RoB:** Some concerns<br>**Average RoB:** Some concerns (applied)<br>**Highest RoB:** Some concerns	Some concerns ✓<br>**Evidence:** mixed<br>**Source:** manual	Some concerns ✓	Major concerns ✓<br>**NMA effect:** 2.000<br>**95% CI:** (-3.259, 7.259)<br>crosses 2 clinically important line(s)<br>**Clinically important:** (-3, 3)	No concerns<br>**NMA effect:** 2.000<br>**95% CI:** (-3.259, 7.259)<br>**95% PrI:** (-3.259, 7.259)	Some concerns ✓<br>**Evidence:** indirect only<br>IF not applicable; judged from the design-by-treatment test.	Very low	| Within-study bias | Reporting bias | Indirectness | Imprecision | Incoherence |

Comparison	Number of Studies	Within-study bias	Reporting bias	Indirectness	Imprecision	Heterogeneity	Incoherence	Confidence rating	Reason(s) for downgrading
**LFD vs MD+LFD+PEN**	--	Some concerns ☑<br>**Majority RoB:** Some concerns<br>**Average RoB:** Some concerns (applied)<br>**Highest RoB:** Some concerns	Some concerns ☑<br>**Evidence:** mixed<br>**Source:** manual	Some concerns ☑	Some concerns ☑<br>**NMA effect:** -0.379<br>**95% CI:** (-3.669, 2.912)<br>crosses 1 clinically important line(s)<br>**Clinically important:** (-3, 3)	No concerns<br>**NMA effect:** -0.379<br>**95% CI:** (-3.669, 2.912)<br>**95% PrI:** (-3.669, 2.912)	Some concerns ☑<br>**Evidence:** indirect only<br>IF not applicable; judged from the design-by-treatment test.	Very low	| Within-study bias | Reporting bias | Indirectness | Imprecision | Incoherence |
