# Supplementary material for: Comparative efficacy of dietary interventions and enteral nutrition in adults with ulcerative colitis: a network meta-analysis
Source: Front Nutr. 2026 Aug 25;13:1886706. doi: 10.3389/fnut.2026.1886706 (PMC13549872; doi:10.3389/fnut.2026.1886706)
Supplement: Supplementary file 2 [file Table_1.DOCX]

| **Database and search details** | **Complete electronic search strategy** |
| --- | --- |
| **PubMed**  **Coverage:** Inception to January 2026  **Records retrieved:** 533 | #1 ("Colitis, Ulcerative"[Mesh] OR "ulcerative colitis"[Title/Abstract] OR "colitis ulcerosa"[Title/Abstract] OR UC[Title/Abstract])  #2 ("Diet Therapy"[Mesh] OR "Nutritional Support"[Mesh] OR "Enteral Nutrition"[Mesh] OR "Parenteral Nutrition"[Mesh] OR diet*[Title/Abstract] OR nutrition*[Title/Abstract] OR "nutritional support"[Title/Abstract] OR "diet therapy"[Title/Abstract] OR "dietary therapy"[Title/Abstract] OR "enteral nutrition"[Title/Abstract] OR "parenteral nutrition"[Title/Abstract] OR "partial enteral nutrition"[Title/Abstract] OR "Mediterranean diet"[Title/Abstract] OR "low FODMAP"[Title/Abstract] OR "low-FODMAP"[Title/Abstract] OR "gluten-free diet"[Title/Abstract] OR "gluten free diet"[Title/Abstract] OR "specific carbohydrate diet"[Title/Abstract] OR "low-residue diet"[Title/Abstract] OR "low residue diet"[Title/Abstract] OR "exclusion diet"[Title/Abstract] OR "IgG-guided"[Title/Abstract] OR "IgG guided"[Title/Abstract] OR "traditional Chinese medicine diet"[Title/Abstract] OR "Chinese medicinal diet"[Title/Abstract])  #3 ("Randomized Controlled Trial"[Publication Type] OR "Controlled Clinical Trial"[Publication Type] OR randomized[Title/Abstract] OR randomised[Title/Abstract] OR randomly[Title/Abstract] OR trial[Title/Abstract] OR placebo[Title/Abstract] OR "controlled trial"[Title/Abstract])  #4 #1 AND #2 AND #3 |
| **Embase**  **Coverage:** Inception to January 2026  **Records retrieved:** 562 | #1 ('ulcerative colitis'/exp OR 'ulcerative colitis':ti,ab,kw OR 'colitis ulcerosa':ti,ab,kw OR uc:ti,ab,kw)  #2 ('diet therapy'/exp OR 'nutritional support'/exp OR 'enteral nutrition'/exp OR 'parenteral nutrition'/exp OR diet*:ti,ab,kw OR nutrition*:ti,ab,kw OR 'nutritional support':ti,ab,kw OR 'diet therapy':ti,ab,kw OR 'dietary therapy':ti,ab,kw OR 'enteral nutrition':ti,ab,kw OR 'parenteral nutrition':ti,ab,kw OR 'partial enteral nutrition':ti,ab,kw OR 'mediterranean diet':ti,ab,kw OR 'low fodmap':ti,ab,kw OR 'low-fodmap':ti,ab,kw OR 'gluten-free diet':ti,ab,kw OR 'gluten free diet':ti,ab,kw OR 'specific carbohydrate diet':ti,ab,kw OR 'low-residue diet':ti,ab,kw OR 'low residue diet':ti,ab,kw OR 'exclusion diet':ti,ab,kw OR 'igg-guided':ti,ab,kw OR 'igg guided':ti,ab,kw OR 'traditional chinese medicine diet':ti,ab,kw OR 'chinese medicinal diet':ti,ab,kw)  #3 ('randomized controlled trial'/exp OR 'controlled clinical trial'/exp OR random*:ti,ab,kw OR trial:ti,ab,kw OR placebo:ti,ab,kw OR 'controlled trial':ti,ab,kw)  #4 #1 AND #2 AND #3 |
| **Web of Science**  **Coverage:** Inception to January 2026  **Records retrieved:** 1442 | #1 TS=("ulcerative colitis" OR "colitis ulcerosa" OR UC)  #2 TS=(diet* OR nutrition* OR "nutritional support" OR "diet therapy" OR "dietary therapy" OR "enteral nutrition" OR "parenteral nutrition" OR "partial enteral nutrition" OR "Mediterranean diet" OR "low FODMAP" OR "low-FODMAP" OR "gluten-free diet" OR "gluten free diet" OR "specific carbohydrate diet" OR "low-residue diet" OR "low residue diet" OR "exclusion diet" OR "IgG-guided" OR "IgG guided" OR "traditional Chinese medicine diet" OR "Chinese medicinal diet")  #3 TS=(random* OR trial OR placebo OR "controlled trial" OR "randomized controlled trial" OR "randomised controlled trial")  #4 #1 AND #2 AND #3 |
| **CNKI**  **Coverage:** Inception to January 2026  **Records retrieved:** 25 | #1 TKA=(溃疡性结肠炎 OR UC)  #2 TKA=(饮食 OR 膳食 OR 营养 OR 营养支持 OR 饮食疗法 OR 膳食干预 OR 肠内营养 OR 肠外营养 OR 部分肠内营养 OR 地中海饮食 OR 低FODMAP OR 低FODMAP饮食 OR 低发酵性寡糖双糖单糖多元醇饮食 OR 无麸质饮食 OR 特定碳水化合物饮食 OR 低渣饮食 OR 排除饮食 OR IgG指导排除饮食 OR 中医食疗 OR 药膳 OR 中医药膳)  #3 TKA=(随机 OR 随机对照 OR 随机对照试验 OR 对照试验 OR 临床试验)  #4 #1 AND #2 AND #3 |
| **SinoMed**  **Coverage:** Inception to January 2026  **Records retrieved:** 562 | #1 主题词/关键词: (溃疡性结肠炎 OR UC)  #2 主题词/关键词: (饮食 OR 膳食 OR 营养 OR 营养支持 OR 饮食疗法 OR 膳食干预 OR 肠内营养 OR 肠外营养 OR 部分肠内营养 OR 地中海饮食 OR 低FODMAP OR 低FODMAP饮食 OR 低发酵性寡糖双糖单糖多元醇饮食 OR 无麸质饮食 OR 特定碳水化合物饮食 OR 低渣饮食 OR 排除饮食 OR IgG指导排除饮食 OR 中医食疗 OR 药膳 OR 中医药膳)  #3 主题词/关键词: (随机 OR 随机对照 OR 随机对照试验 OR 对照试验 OR 临床试验)  #4 #1 AND #2 AND #3 |
| **Wanfang**  **Coverage:** Inception to January 2026  **Records retrieved:** 1167 | #1 主题: (溃疡性结肠炎 OR UC)  #2 主题: (饮食 OR 膳食 OR 营养 OR 营养支持 OR 饮食疗法 OR 膳食干预 OR 肠内营养 OR 肠外营养 OR 部分肠内营养 OR 地中海饮食 OR 低FODMAP OR 低FODMAP饮食 OR 低发酵性寡糖双糖单糖多元醇饮食 OR 无麸质饮食 OR 特定碳水化合物饮食 OR 低渣饮食 OR 排除饮食 OR IgG指导排除饮食 OR 中医食疗 OR 药膳 OR 中医药膳)  #3 主题: (随机 OR 随机对照 OR 随机对照试验 OR 对照试验 OR 临床试验)  #4 #1 AND #2 AND #3 |
