# Supplementary material for: Comparative efficacy of dietary interventions and enteral nutrition in adults with ulcerative colitis: a network meta-analysis
Source: Front Nutr. 2026 Aug 25;13:1886706. doi: 10.3389/fnut.2026.1886706 (PMC13549872; doi:10.3389/fnut.2026.1886706)
Supplement: Supplementary file 3 [file Table_4.DOCX]

**PRISMA-NMA Checklist**

| **Section** | **Topic** | **Item #** | **PRISMA-NMA checklist item** | **Reported location / manuscript evidence** | **Status / recommended action** |
| --- | --- | --- | --- | --- | --- |
| TITLE | Title | 1 | Identify the report as a systematic review incorporating a network meta-analysis. | Title: “Comparative Efficacy of Dietary Interventions and Enteral Nutrition in Adults with Ulcerative Colitis: A Network Meta-Analysis”. | Reported |
| ABSTRACT | Structured summary | 2 | Structured summary: background/objectives; methods; results; conclusions; funding and registration where applicable. | Abstract reports Objective, Methods, Results, and Conclusion; databases/search date; RCT eligibility; Bayesian NMA; RoB 2/CINeMA; 14 trials (1,039 participants; 10 interventions); main estimates, confidence ratings, and a cautious conclusion. Registration and funding are absent from the Abstract. | Partly reported; add registration and funding if journal format permits |
| INTRODUCTION | Rationale | 3 | Describe rationale for the review, including why NMA was conducted. | Introduction paragraphs 1–4 describe UC burden, limitations of drug therapy, the rationale for dietary/nutritional strategies, gaps in previous reviews, and the need to compare multiple interventions by NMA. | Reported |
| INTRODUCTION | Objectives | 4 | Explicit statement of review question with PICOS. | The final Introduction paragraph states the aim of comparing dietary and nutritional interventions in adults with UC; PICOS elements are specified in Methods 2.2. | Reported |
| METHODS | Protocol and registration | 5 | Indicate whether protocol exists and provide registration information. | Methods 2.1 reports prospective PROSPERO registration (CRD420251076943) and adherence to the PRISMA extension for network meta-analysis. | Reported |
| METHODS | Eligibility criteria | 6 | Specify PICOS, follow-up/report characteristics, eligible treatments, clustering/merged nodes if any. | Methods 2.2 specifies adults ≥18 years with confirmed UC; structured dietary or enteral nutrition interventions; alternative diet/standard diet comparators; outcomes; RCT design; Chinese/English reports; and exclusions. Results 3.2 lists 10 intervention nodes. | Reported; clarify that no treatment nodes were merged, if accurate |
| METHODS | Information sources | 7 | Describe information sources and date last searched. | Methods 2.3 reports six databases searched from inception through January 2026 and reference-list screening of included studies and related reviews. | Reported |
| METHODS | Search | 8 | Present full electronic search strategy for at least one database. | Methods 2.3 states that the full electronic search strategy is provided in Supplementary Docx 1. | Reported if Supplementary Docx 1 contains a reproducible database strategy |
| METHODS | Study selection | 9 | State process for selecting studies. | Methods 2.4 reports EndNote 21 deduplication, independent screening by two reviewers, and resolution by discussion or a third reviewer. Results 3.1 reports selection counts. | Reported |
| METHODS | Data collection process | 10 | Describe data extraction method and confirmation processes. | Methods 2.4 reports independent data extraction by two reviewers using a standardized Excel spreadsheet, with disagreements resolved by discussion or a third reviewer. | Reported |
| METHODS | Data items | 11 | List and define variables sought and assumptions/simplifications. | Methods 2.4 lists author, year, sample size, age, duration, intervention characteristics, and outcomes. Methods 2.2 defines outcomes; Supplementary Table S1 summarizes effect modifiers. | Reported; assumptions/simplifications could be stated more explicitly |
| METHODS | Geometry of the network | S1 | Describe methods used to explore network geometry and potential related biases. | Methods 2.7 states that networks were visualized in Stata 18.0; Results 3.4.1/Figure 3 present four outcome networks. Node/edge scaling and planned geometry-bias assessment are not explicitly described. | Partly reported; add node/edge definitions and geometry-assessment methods |
| METHODS | Risk of bias within individual studies | 12 | Describe methods for assessing risk of bias of individual studies and use in synthesis. | Methods 2.5 describes RoB 2, five domains, two independent reviewers, and third-reviewer adjudication. High-overall-risk studies were excluded in sensitivity analyses. | Reported |
| METHODS | Summary measures | 13 | State principal summary measures and ranking measures. | Methods 2.7 specifies ORs for dichotomous outcomes and MDs for continuous outcomes, each with 95% credible intervals, and SUCRA values for treatment rankings. | Reported |
| METHODS | Planned methods of analysis | 14 | Describe data handling and methods for each NMA, including multi-arm trials, variance structure, priors, model fit. | Methods 2.7 reports random-effects consistency models; binomial-logit and normal-identity models; four MCMC chains; 20,000 tuning and 100,000 simulation iterations; thinning 10; variance scaling 2.5; 20,000 inference samples; and Brooks–Gelman–Rubin convergence. Priors and multi-arm handling are not stated. | Partly reported; specify priors and multi-arm handling, if applicable |
| METHODS | Assessment of inconsistency | S2 | Describe statistical methods to evaluate agreement of direct and indirect evidence and actions if inconsistency found. | Methods 2.7 states that no network contained a closed loop; formal direct-versus-indirect inconsistency assessment was therefore not feasible. | Reported as not feasible |
| METHODS | Risk of bias across studies | 15 | Specify assessment of publication bias/selective reporting across studies. | Methods 2.7 specifies comparison-adjusted funnel plots when feasible. Results 3.5 reports plots for four outcomes and Egger-type tests where estimable, with caution because each outcome included fewer than 10 studies. | Reported |
| METHODS | Additional analyses | 16 | Describe additional analyses if done and whether prespecified. | Methods 2.7 describes sensitivity analyses excluding high-risk studies or studies with <30 participants when connectivity was retained. Disease-severity subgroup analysis was not feasible because subgroup data were unavailable. | Reported; whether analyses were prespecified is not stated |
| RESULTS | Study selection | 17 | Give numbers screened, assessed, included, reasons for exclusion; ideally flow diagram. | Results 3.1 reports 4,291 records identified; 998 duplicates removed; 3,293 screened; 2,723 excluded; 570 full texts assessed; 556 excluded with reasons; and 14 included. Figure 1 is the PRISMA flow diagram. | Reported |
| RESULTS | Presentation of network structure | S3 | Provide network graph. | Results 3.4.1 and Figure 3 provide network maps for clinical effectiveness, ALB, IBDQ, and CRP. | Reported |
| RESULTS | Summary of network geometry | S4 | Brief overview of network characteristics, gaps, abundance of trials/patients, potential network bias. | Results 3.4.1 reports 14 studies, 10 interventions, and 6 outcomes; FCP and SIBDQ each had one study and were excluded from NMA. Study counts per network are reported; the Discussion notes sparse networks and no closed loops. | Reported |
| RESULTS | Study characteristics | 18 | Present extracted characteristics for each study with citations. | Results 3.2/Table 1 present 14 studies (1,039 participants): disease stage, intervention/comparator, sample size, sex, age, duration, and outcomes. Supplementary Table S1 summarizes effect modifiers and concomitant therapy. | Reported |
| RESULTS | Risk of bias within studies | 19 | Present risk-of-bias data for each study/outcome. | Results 3.3 and Figure 2 present domain-level and overall RoB 2 judgments; 12 studies had some concerns and 2 were at high risk. | Reported |
| RESULTS | Results of individual studies | 20 | For all outcomes, present group summary data and effect estimates with CIs. | Table 1 presents study characteristics and outcome availability; Tables 2–5 present network estimates. Arm-level outcome data and individual-study effect estimates are not fully reported. | Partly reported; add arm-level data or individual-study estimates in a supplement |
| RESULTS | Synthesis of results | 21 | Present results of each meta-analysis including intervals; ranking measures if explored. | Results 3.4.2–3.4.7 and Tables 2–7 report ORs/MDs with 95% credible intervals, SUCRA rankings, and CINeMA confidence ratings. | Reported |
| RESULTS | Exploration for inconsistency | S5 | Describe results from inconsistency investigations. | Methods 2.7 and Discussion 4.3 state that no closed loops were present, so formal inconsistency assessment between direct and indirect evidence was not feasible. | Reported as not feasible |
| RESULTS | Risk of bias across studies | 22 | Present results of any across-study bias assessment. | Results 3.5 presents funnel plots for four outcomes. Egger-type tests were non-significant for clinical effectiveness (0.37, p=0.26), ALB (0.56, p=0.16), and CRP (−0.10, p=0.97); IBDQ was not estimable. Publication bias cannot be excluded. | Reported |
| RESULTS | Results of additional analyses | 23 | Give results of additional analyses if done. | Results 3.4.8 reports sensitivity analyses. EN versus LRD remained significant after excluding high-risk and very small studies; CRP findings were unchanged. ALB/IBDQ analyses were infeasible because networks disconnected. | Reported |
| DISCUSSION | Summary of evidence | 24 | Summarize main findings and strength/relevance of evidence. | Discussion 4.1 summarizes EN versus LRD for clinical effectiveness; EN, IgG-ED, LFD, and RD versus LRD for ALB; and no significant IBDQ/CRP differences. SUCRA rankings are exploratory, and all estimates have low/very low confidence. | Reported |
| DISCUSSION | Limitations | 25 | Discuss study/outcome and review-level limitations; transitivity, consistency, network geometry concerns. | Discussion 4.3 addresses sparse evidence, wide intervals, threats to transitivity from effect-modifier imbalance, incomplete background-therapy reporting, absent closed loops, low/very low confidence, and heterogeneous outcome definitions. | Reported |
| DISCUSSION | Conclusions | 26 | Provide interpretation in context and implications for future research. | Discussion 4.4 frames dietary/enteral nutrition interventions as individualized adjuncts and identifies future research priorities. Section 5 gives a cautious conclusion and calls for larger rigorous RCTs. | Reported |
| FUNDING | Funding | 27 | Describe sources of funding/support and funder role; conflicts related to treatments. | The Funding section lists three grants: Guangdong Provincial Administration of Traditional Chinese Medicine (20261006), Jiangsu Provincial Traditional Chinese Medicine Science and Technology Development Program (MS2025043), and China Hospital Reform and Development Research Institute/Nanjing Drum Tower Hospital (NDYG2025020). Funder roles are not stated; conflicts are reported separately. | Partly reported; add a statement on funders’ roles |
